# Follow-Up of Offspring Born to Parents With a Solid Organ Transplantation: A Systematic Review

**DOI:** 10.3389/ti.2022.10565

**Published:** 2022-08-05

**Authors:** Jildau R. Meinderts, Jelmer R. Prins, Stefan P. Berger, Margriet F. C. De Jong

**Affiliations:** ^1^ Department of Nephrology, University Medical Center Groningen, Groningen, Netherlands; ^2^ Department of Obstetrics and Gynecology, University Medical Center Groningen, Groningen, Netherlands

**Keywords:** transplantation, long-term, offspring, follow-up, pregnancy

## Abstract

Pregnancy after solid organ transplantation (SOT) has potential risks for the offspring. Most existing research focused on short-term pregnancy outcomes. The aim of this systematic review was to evaluate available data concerning longer term outcomes (>1 year) of these children. A systematic literature search, following PRISMA guidelines, of PubMed and Embase was performed from the earliest date of inception through to 6th April 2022. Publications on all types of (combined) SOT were eligible for inclusion. In total, 53 articles were included. The majority assessed offspring after kidney (78% of offspring) or liver transplantation (17% of offspring). 33 studies included offspring aged >4 years and five offspring aged >18 years. One study was included on fathers with SOT. The majority of the 1,664 included children after maternal SOT had normal intellectual, psychomotor, and behavioral development. Although prematurity and low birth weight were commonly present, regular growth after 1 year of age was described. No studies reported opportunistic or chronic infections or abnormal response to vaccinations. In general, pregnancy after SOT appears to have reassuring longer term outcomes for the offspring. However, existing information is predominantly limited to studies with young children. Longer prospective studies with follow-up into adulthood of these children are warranted.

## GRAPHICAL ABSTRACT

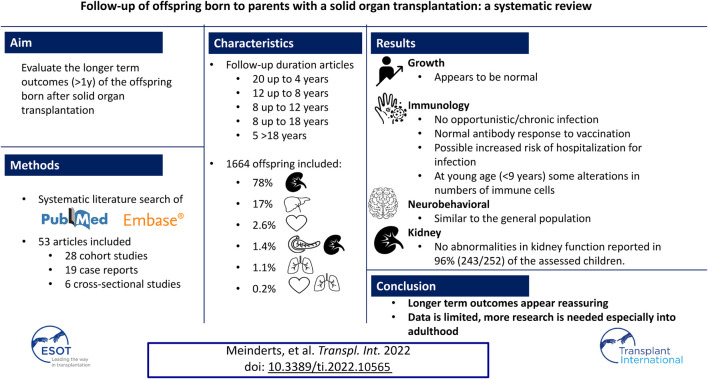

## Introduction

Solid organ transplantations (SOT) are increasingly performed worldwide. Pregnancy numbers after SOT have increased. Over 3,200 pregnancies after maternal SOT have been described in the Transplant Pregnancy Registry International (TPRI) database (1). SOT pregnancies are associated with increased incidence of prematurity and low birth weight (LBW) (1–3). All pregnancies after SOT are classified as high risk (1), but risk differs per SOT. The most severe risk is seen after heart and lung transplantation (HTx, LuTx) (1). However, after kidney and liver transplantation (KTx, LiTx), live birth rate and miscarriage rates are reported to be similar to the general population (3–5), and the majority of offspring in SOT pregnancies are reported as healthy at birth (1–4). Most data on the offspring only focused on perinatal outcomes such as prematurity, birth weight, congenital abnormalities, congenital infections, and APGAR scores. A recent overview on post-transplant pregnancy by Klein et al. emphasized the lack of available data on the long-term health of the offspring (6). To the best of our knowledge, no systematic review on longer term outcomes after birth of the offspring born after SOT exists. Therefore, the aim of this systematic review is to evaluate the available data concerning longer term outcomes (>1 year) of children of SOT patients.

## Materials and Methods

### Data Sources and Searches

A systematic literature search, made in consultation with an information specialist, of PubMed and Embase was performed, from the earliest date of inception through to 6th April 2022. A protocol for the systematic review was prepared locally but not submitted or registered online. The following key terms and their synonyms were used: organ transplantation (with all SOT transplants: heart, lung, kidney, liver, pancreas and small bowel separately mentioned), pregnancy, child. A reproducible search strategy is provided in Supplementary Table S1.

### Study Selection

All pregnancies with either a mother or father with a SOT (heart, lung, kidney, liver, pancreas, or small bowel) as well as combined SOT in their history were eligible for inclusion. Articles were included if >1-year follow-up data of the offspring was described. In overlapping articles, the most recent article was included. Articles not written in English, conference abstracts, (systematic) reviews, and meta-analysis were excluded.

**FIGURE 1:**
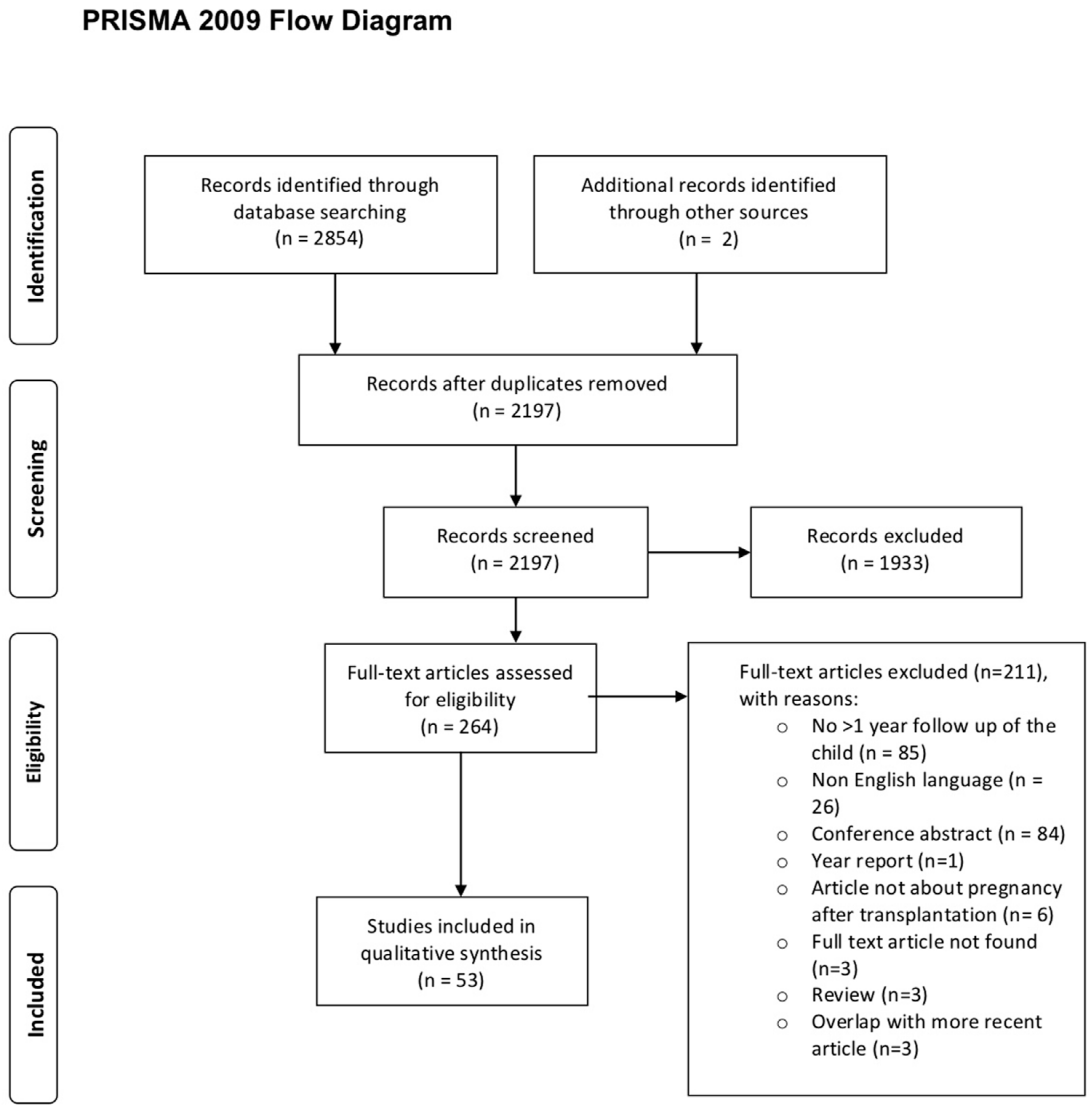
PRISMA flow diagram study inclusion.

Initial selection based on title and abstract was performed by two researchers (JRM and MFC) independently. All disagreements were discussed and, if there was doubt, the study was included for full-text screening, performed by the same two researchers. All discrepancies during full-text screening were resolved by consensus by the same two researchers. All citations of eligible articles and relevant review articles were consulted for Supplementary References. Two articles were identified that were not found in the primary search. The PRISMA (preferred reporting items for systematic review and meta-analyses) flowchart (7) was used to document the number of articles included and excluded, including the rationale for exclusion (Figure 1).

### Data Extraction

Data extraction was carried out by one researcher (JRM). A second researcher (MFC) independently performed a full-text check for accuracy and completeness. All discrepancies were resolved by consensus of the authors. For each included study the following data was extracted and summarized in two tables: first author, country, study type, follow-up period, number of live births, transplanted organ, immunosuppressive regimen, mean/median birth weight, mean/median gestational age, method of assessment, and the longer-term outcomes. All longer-term outcomes were evaluated, with specific attention for growth, immunological, neurocognitive, and behavioral follow-up and kidney function. Data on intra uterine fetal deaths and miscarriages were not included. Authors of primary studies were not contacted to provide missing data. Biased appraisal of the articles was performed by two researchers (JRM and JRP) (Supplementary Tables S2A,S2B). For prospective and retrospective cohort studies we used the Newcastle Ottawa Scale (NOS) for cohort studies (8). For cross-sectional studies and case reports we used the applicable Joanna Briggs Institute (JBI) critical appraisal checklists (9).

### Definitions

The following definitions were used: preterm: <37 weeks of gestation, LBW: <2,500 g, and catch-up growth: rapid growth in children following a period of reduced growth (10).

## Results

The systematic search yielded 2,854 articles. 657 were duplicates (Figure 1). After full text screening (*n* = 264), *n* = 53 articles were selected (Tables 1, 2; Supplementary Tables S2A,S2B), yielding 19 case reports, 18 retrospective, 10 prospective, and six cross-sectional cohort studies. In 16 studies a comparison with a control group was made, whereby the control group was matched in 12 studies. In 13 articles, pregnancies after multiple SOT types were assessed, leading to 36 articles assessing offspring born after KTx, 16 after LiTx, three after combined pancreas-kidney transplantation, seven after HTx, one after LuTx, and one after combined heart-lung transplantation. No article about offspring born after small bowel transplantation was found. One study on children from fathers with SOT was found (11). The majority of the studies (*n* = 33 (62%)) reported results on children with a follow-up of >4 years. Of these, twelve reported on children aged up to 8 years and eight on children aged up to 12 years. Follow-up on children aged up to 18 years was reported in eight studies and in five studies, children above the age of 18 were included (Tables 1, 2). Paragraphs 3.1–3.6 describe the offspring born to mothers with a SOT; paragraph 3.7 describes the offspring born to a father with a SOT.

**TABLE 1:** Included studies, cohort studies.

Author (Year), country	Transplanted organ, number of children	Follow-up age children	Outcome measures
Devresse (2022), Belgium (13)	Kidney: 43 infants (2 twins) from 32 women (57 pregnancies), 48% female	Median follow-up 17 years (range 7–25)	• Questionnaire sent to 43 children or their parents if < 18 years. 21 responded. Questions on current situation (weight, height, familial status, and treatment), medical history (hypertension, diabetes, and depression), addictions (smoking, etc.) and education
Egerup (2021), Denmark (49)	Kidney: 124 infants	Median follow-up 14.5 years [IQR 7.1–22.8]	• Administrative codes of diagnosis and antibiotic prescriptions identified in national registries
	Control: 1,231 infants	Median follow-up control group 14.1 years IQR 6.6–25.4]	
Borek-Dziecioł (2020), Poland (58)	Kidney: 40 infants	Newborns, infants, and children over 1 year of age were examined. Not described at what age	• Renal parameters: urea, creatinine, potassium, and sodium concentration were analyzed
	Control: 40 infants		
Dębska-Slizien (2020), Poland (26)	Kidney: 25 infants	Median follow-up 9 years (range 0.5–30 years)	• No specific long-term outcomes described
Bachmann (2019), Germany (40)	Kidney: 30 infants	Follow-up at birth, 12 and 24 months 65.6% of the children had a complete dataset at 24 months	• Physical and psychomotor development examination by a pediatrician, collected from the patient file (weight, length, and head circumference)
	Combined kidney-pancreas: 2 infants		• Questionnaire filled in by the mother about the child (physical examination, anthropometric measures, medical, and paramedical history)
Morales-Buenrostro (2019), Mexico (50)	Kidney: 50 infants	Children >4 years were included. Most children were aged between 6 and 16 years (*n* = 32 in the study group and *n* = 37 in the control group)	• Interview with the mother and the child
	Control: 50 infants		• Intellectual performance: IQ scores (age-specific test: WPPSI, WISC-IV, WAIS-III)
Schreiber-Zamora (2019), Poland (1) (51)	Kidney: 36 infants	Follow-up at one time-point, median 3.12 years	• Age-specific neurological examination including ultrasound
	Control: 36 infants		
Schreiber-Zamora (2019), Poland (2) (42)	Liver: 35 infants	Follow-up Tx group: 6 children 1–12 months, 15 children 1–3 years, 25 children 3–6 years, 15 children >6 years	• Measurement of BMI as a one-time measurement
	Kidney: 26 infants	Follow-up control group: 7 children 1–12 months, 16 children 1–3 years, 24 3–6 years, 17 children >6 years	
	Control: 64 infants		
Turkyilmaz (2018), Turkey (14)	Liver: 8 infants	Mean follow-up 3.2 years ± 2.4 years, range 1–7 years	• Retrospective analyses of patient records, no specific long term outcome measurements described
Kociszewska-Najman (2018), Poland (52)	Liver: 42 infants	1 assessment per child (*n* = 31 < 30 months, *n* = 47 > 30 months)	• Psychological examination performed by qualified clinical psychologists. Results expressed in IQ (age specific tests: Psyche Cattell Infant Intelligence Scale, Terman-Merril Intelligence Scale, Scales of Raven’s Progressive Matrices)
	Kidney: 38 infants		
	Control: 78 infants		
Ono (2015), Brazil (44)	Kidney: 28 infants (1 twin)	Immunological follow-up at birth and at 8 months of age. General follow-up by the pediatrician every month during the first 6 months, every 3 months until 2 years of age	• Blood sample collection at birth from the umbilical cord and at 8 months from a peripheral vein. Immuno-phenotypic studies were done with fresh blood. Each sample was stained with fluorochrome-conjugated monoclonal antibodies
	Control group 1: 40 infants		• Factors associated with hospital admission were analyzed by univariate logistic regression
	Control group 2: 28 infants		
Czaplinska (2014), Poland (60)	Liver: 51 infants	Neonates, infants, and children >1 year of age were examined. Not described at what age	• Analysis of liver parameters: alanine transaminase (ALT) and aspartate transaminase (AST) and two kidney parameters (urea and creatinine)
	Control: 51 infants		
Norrman (2014), Sweden (61)	Kidney	Group 1: mean age at follow-up: 9.7 ± 4.2 years	• Retrospective analyses of 5 registries: National Quality Register of Assisted Reproduction, the National Register in IVF, the Swedish Medical Birth Register, the National Patient Register, and the Swedish Cause of Death Register
	Group 1: 7 infants (1 twin)	Group 2: mean age of follow-up: 14.7 ± 9.4 years	
	Group 2: 199 infants		
	Control		
	Group 3: 665 infants		
	Group 4: 3,980 infants		
Drozdowska-Szymczak (2014), Poland (48)	Kidney: 39 infants	Follow-up at 1 time-point, range: 1 day-15 years (*n* = 26 > 10 months) and in the control group 1 day till 14 years	• Serum IgG and IgM measurements at 1 time-point with agglutination immunoassays
	Control: 39 infants		
Kociszewska-Najman (2013), Poland (62)	Liver: 37 infants	Follow-up: neonatal (1–4 weeks of age), babyhood (2–12 months), early kindergarten (1–3 years), later kindergarten (4–6 years) and school years (>6 years). Not all children at all follow-up moments tested. Most children tested in the late kindergarten stage	• Retrospective analyses of the parameters in the neonatal period of the child
	Kidney: 45 infants		• Prospective ophthalmological examinations by a pediatric ophthalmologist
	Control: 66 infants		
Shaner (2012), United States (16)	Lung: 18 infants (1 triplet)	Follow-up mean: 7.0 years (± 5.37), range: 1.25 till 17.36 years	• NTPR registry and retrospective questionnaires, no specific long-term outcomes described
Nulman (2010), Canada (30)	Kidney: 39 infants	Mean follow-up 8.06 years, range: 3 years 7 months till 15 years 9 months	• Physical examination of the child (weight, length, and head circumference)
	Control: 38 infants		• Psychological examination of mother and child conducted by a trained psychologic assistant under supervision of a registered psychologist
			• Child: IQ: WPPSI-R, Visuomotor abilities: VMI-4 and the WRAVMA.
Al-Khader (2004), Saudi-Arabia (12)	Kidney: 110 infants (3 twins)	Follow-up of 41 infants, mean follow-up: 52 months (range: 13–83 months)	• Retrospective analyses of medical records including laboratory measurements, no details on the method of follow-up mentioned
Miniero (2004), Italy (18)	Kidney: 52 infants	Follow-up ranging from 2 months till 13 years	• Retrospective questionnaires, patient record data, and interviews in person or by telephone (growth, vaccinations, allergic reactions, diseases, laboratory tests, and last measured height and weight)
	Liver: 7 infants		
	Heart: 8 infants (1 twin)		
Bar (2003), Israel (63)	Kidney: 48 infants	Follow-up 2–7 years	• Retrospective analyses of medical records (short-term outcomes e.g., caesarean delivery, hospitalization, stillbirths)
	Control: 48 infants		• Blinded periodical examination up to 7 years (maternal renal function, infant status, presence of severe handicap)
Sgro (2002), Canada (31)	Kidney: 32 infants	Follow-up mean 3.1 year (range 3 months till 11 years)	• Retrospective analyses of medical records
	Control: 88 infants		• Pediatric follow-up visit: physical examination including growth parameters, neurodevelopmental assessment (Denver Developmental Screening test)
Giudice (2000), France (32)	Kidney: 10 infants (1 twin)	Follow-up of 12 children at 2.6 ± 1.8 years (range 1.0–6.9 years)	• Renal function tests (blood pressure, inulin clearance, paraminohippuric acid clearance, microalbuminuria, electrolyte reabsorption rate, renal ultrasound including renal size)
	Pancreas-kidney: 1 infant		• Retrospective neonatal history
	Heart: 2 infants		• Complete physical examination at the time of the renal function study
	Liver: 1 infant		
Willis (2000), United Kingdom (33)	Kidney: 48 infants (1 triplet)	Median follow-up: 5.2 years (range 9 months–18 years)	• Surveys, semi-structured interviews, medical records, and physical examination carried out by a researcher (blood pressure, developmental milestones, scholastic and educational achievements, urine sample, ultrasound examination of the urinary tract)
Stanley (1999), United States (56)	Kidney: 175 infants (52% girls)	Range of the child’s age at interview: 4 months-12 years, mean age: 4.4 years	• Assessment of developmental status (≤5 years: Child Development Review system, >5 years: prior developmental or present educational morbidity reported by the mother)
McGrory (1998), United States (19)	Combined pancreas -kidney and 1 pancreas followed by kidney: 20 infants	Follow-up ranging from 1 month to 8 years	• Data collected from a questionnaire, medical records, and telephone interviews. No specific long-term outcome measurements
Wu (1998), Germany (34)	Liver: 23 infants (1 twin)	Follow-up range 1–99 months 5 children <1 year at last follow-up	• Data obtained via medical records and questionnaires evaluated by the pediatrician (height and weight, psychological development, neurological development)
Jain (1997), United States (35)	Liver: 27 infants (long-term follow-up *n* = 25)	Multiple, frequency and timing not specified, follow-up moments. Median follow-up of 39 months (range 10–76 months)	• Prospectively collected data by patients, obstetricians, and the physicians. Weight for age percentiles calculated from the National Center for Health Statistics percentiles
Wong (1995), New-Zealand (55)	Kidney: 11 infants	Follow-up ranging from 15 months to 18 years	• Retrospective information from medical records (clinical and laboratory data, physical growth, physical examination, school performance, work achievement, social behavior, developmental milestones tested with the Denver developmental screening test)
Pilarski (1994), Canada (45)	Kidney: 11 infants	1 follow-up per infant. Follow-up time ranging from 5 months till 9 years (1 child <1 year at follow-up)	• Immunological assessment of blood samples
	Liver: 1 infant		
Pahl (1993), United States (43)	Kidney: 26 infants	Mean follow-up: 5 years, range: 1 week - 18 years (5 children <1 year)	• Analyses of medical records (mother and child if present), interviews with the physician, interviews of the mothers by telephone or email (childhood development of their child (ren))
Shaheen (1993), Saudi Arabia (59)	Kidney: 26 infants	Mean follow-up 39 months (range 6–72 months)	• Basic tests of kidney function and integrity on 22 children
			• Serum cyclosporine was measured in whole blood using radioimmunoassays
Wagoner (1993), United States (25)	Heart: 28 infants	Mean follow-up 3.4 years (range 3 months till 6.5 years)	• Questionnaires study: no specific long-term outcome measurements described
	Heart and lung: 3 infants		
Rasmussen (1981), Sweden (47)	Kidney: 5 infants	Follow-up ranging from 4.5 to 9 years. Follow-up frequency between 2 and 4 times	• Somatic and psychomotor evaluation at regular intervals
			• Immunological follow-up from peripheral blood at multiple time points: % rosette-forming PBM’s, proliferative responses of PBM to phythemagglutinin and pokeweed mitogen, counting the PBMs with surface immunoglobulins using fluoresceinated anti-light chain antisera, quantitative immunoglobulin levels for IgG, IgA and IgM, serum testing for antibodies against hepatitis B, polio virus, Haemophilus influenza, and *Escherichia coli*. Serum aspartate transferase and alanine transferase in HBsAg-positive children
			• Chromosomal analyses performed in 4 children
Korsch (1980), United States (11)	Fathers with a kidney Tx: 4 infants (0 girls) from 3 fathers	Follow-up: ranging from 4 months to 6 years and 8 months (father KT: 4 months, 10 months, 11 months, 2 years 7 months and mother KT: 7 months, 1 year, 1 year 10 months, 2 years 4 months, 3 years 2 months, 6 years 8 months)	• Patient records, physical examination by a pediatrician
	Mothers with a kidney Tx: 6 infants (2 girls) in 5 women		• Developmental evaluations on nine of the children by a specialist in assessing child development (age specific: Stanford-Binet test, Gesell Developmental Schedules, and Bayley Scales of Infant Development)
			• A semi-structured interview by a social work assistant trained in sociologic research methods on the parents’ attitudes about their child’s development

**TABLE 2:** Included studies, case reports.

Author (Year), country	Transplanted organ, number of children	Follow-up age children	Outcome measures
Rao (2019), Australia (41)	Kidney: 1 infant	Follow-up 2 years	• The weight of the infant was followed up for 2 years
Mahmoud (2017), Kuwait (15)	Kidney: 4 infants (1 triplet)	Follow-up at birth, discharge, 12 months and 24 months	• No specific outcome measurements described
Kociszewska-Najman (2012), Poland (29)	Liver: 2 infants	1 infant: follow-up visit at 7 months 1 child follow-up visit at 21 months	• Length, weight, head circumference, blood pressure, laboratory tests, abdominal ultrasound, and echocardiogram
			• Neurodevelopmental and socio-emotional assessment
			• Mental ability tested with the Cattell Infant Intelligence scale
Nicovani (2009), Chile (27)	Kidney: 3 infants (triplet)	4 years follow-up	• No specific long term outcome measures described
Xia (2008), China (17)	Liver: 1 infant	Follow-up 4 years, every 3–6 months	• Routine follow-up visits, patient self-examination of the baby’s growth and development
Scott (2002), United States (28)	Kidney: 5 infants (3 girls) (1 mother)	Follow-up at one time-point, age of the offspring: 23, 21, 18, 17, 15 years	• No outcome measurements described
Morini (1998), Italy (20)	Heart: 1 infant	Follow-up 14 months	• No specific long-term outcome measurements
Roll (1997), Germany (36)	Liver: 1 infant	Follow-up of 2 years and 6 months	• No specific long-term outcome measurements
Eskandar (1996), Canada (21)	Heart: 2 infants	Follow-up of >2 years in both children	• No specific long-term outcome measurements
Morita (1996), Japan (37)	Kidney: 8 infants	Mean follow-up: 4.1 years (range: 1 year till 11 years)	• 1-time point of evaluation. No specific method of assessment mentioned
Liljestrand (1993), Sweden (64)	Heart: 1 infant	Follow-up 18 months	• Specific long-term outcome measurements not described
			• At 12 months: detailed evaluation at a regional specialized center in pediatric cardiology
Baarsma (1992), Netherlands (46)	Liver: 1 infant	Follow-up 2-year, not clear how many follow-up moments	• Immunological assessment of blood samples and functional assessment of the immune system
Grow (1991), United States (54)	Liver: 2 infants (twins)	Neurodevelopmental follow-up of 25 months	• Unspecified neurodevelopmental follow-up
Scantlebury (1990), United States (53)	Liver: 20 infants (1 twin)	Follow-up ranging from 9 months till 12 years (*n* = 16 > 1 year)	• No specific long-term outcome measurements described
Key (1989), United States (22)	Heart: 1 infant	Follow-up of 3 years	• No specific long-term outcome measurements described
Preieto (1989), Spain (23)	Kidney: 4 infants (2 sets of twins)	1 twin follow-up at 22 months and 1 twin at 8 months	• No specific long-term outcome measurements described
Boner (1981), Israel (24)	Kidney: 2 infants (twins)	Follow-up of 6 years	• No specific long-term outcome measurements for the physical and psychological assessment described
			• Cell mediated immunity examination at 8–10 months with blood samples: lymphocytic transformation measurement with phytohemaglutinin, estimation of the secretion of macrophage migration inhibition factor, PPD skin test, delayed hypersensitivity skin tests
Berant (1976), Israel (38)	Kidney: 1 infant	Multiple follow-up visits: at birth, 3 months, 5 months and 2 years	• Immunological evaluation with blood samples
			• At birth: chest x-ray for the thymic shadow
			• Lymphocytic transformation by phytohemagglutinin at birth and 2 years
Price (1976), United Kingdom (39)	Kidney: 2 infants	1 child follow-up of 32 months and 1 child follow-up of 24 months. Not specified how many follow up moments	• No specific long-term outcome measurements described, developmental tests not specified
	Control: 54 infants		• Blood lymphocyte, cortisol levels, and chromosome analyses measured at multiple timepoints

### Characteristics of Included Patients

A total of 1,664 live births were recorded, of which 78% (*n* = 1,290) were born after KTx, 17% (*n* = 287) after LiTx, 2.6% (*n* = 43) after HTx, 1.4% (*n* = 23) after combined pancreas-kidney transplantation, 1.1% (*n* = 18) after LuTx, and 0.2% (*n* = 3) after combined heart-lung transplantation. In pregnancies of which the complete immunosuppressive regimen was known, 78% of the women used corticosteroids, 49% cyclosporine, 41% azathioprine, and 30% tacrolimus. In 51 pregnancies with a live birth mycophenolate mofetil (MMF) was used for at least part of gestation, in five pregnancies rapamycin, and in one pregnancy everolimus. Two studies did not specify if MMF and/or rapamycin was stopped during pregnancy (12, 13). No congenital abnormalities were mentioned in these live-born children. Details on gestational age and birth weight can be found in Supplementary Tables S2A,S2B. In 15 articles (*n* = 191 children, 12%) “normal development,” “no problems,” or “doing well” was mentioned without conducting specific tests or parameters (14–28).

### Growth

Specific results on growth were described in 16 articles (11, 29–43), of which 6 are case reports (29, 36–39, 41). Overall these results indicate that growth development in the offspring of SOT patients is normal. Of the 234 children born after KTx, 219 (94%) had weight and length development comparable to the general population (11, 30–33, 37–40, 42, 43). Sgro et al. reported a significantly higher weight for age and a significantly lower length for age at a mean follow-up of 3.1 years (range 3 months-11 years) in the KTx offspring group compared to the control group (31). Schreiber-Zamora et al. reported no significant differences in the prevalence of overweight and underweight when comparing offspring of KTx recipients (KTR) with offspring of LiTx recipients and a control group. In the transplant group 16.4% had obesity and in the control group 6.3% did (*p* = 0.072). The theoretical incidence of obesity in the general population (5%) was significantly lower than the incidence in the LiTx (17.1%), the KTx offspring (15.4%), and the overall transplant group (16.4%) (*p* < 0.001, *p* = 0.02, *p* < 0.001 respectively). Prenatal exposure to tacrolimus was associated with a 2.8-fold increased risk for developing a higher body mass index at later follow-up (42).

Catch-up growth was reported in three case reports with three children from KTR and one child from a LiTx recipient (36, 38, 39). Willis et al. also reported impressive catch-up growth in 21 children born with a birth weight <10th percentile (33). In five articles the growth of 86 infants born after LiTx was evaluated; even though birth weight was low, subsequent height and weight development was within the normal range (29, 34–36, 42).

### Immunological Follow-Up

Ten studies focused on immunological follow-up of the offspring (18, 29, 38, 39, 44–49), of which four were case reports (29, 38, 39, 46). In none of the included studies were opportunistic or chronic infections reported. Antibody response to vaccination was normal and no side-effects of vaccination were observed (18, 38, 45, 46). Two studies reported a significantly higher number of children hospitalized due to infectious disease in the KTx offspring group compared to a control group (44, 49). Ono et al. reported that 28.6% of the KTx group compared to 7.5% of the unmatched control group was hospitalized (*p* = 0.046) (44). All hospitalized children were exposed to tacrolimus during pregnancy. Egerup et al. matched a KTx offspring group aged 0–5 years with a control group in a 1:10 ratio. 41.9% of the KTx offspring compared to 24.8% of the control group were hospitalized due to infectious disease (risk ratio 1.67). The average number of antibiotic prescriptions filled between age 1–5 years was significantly higher in the KTx compared to the controls. However, this difference was not observed in the group as a whole (age 0–5 years) and not for group <1 year (Supplementray Table S2A) (49).

Serum levels of IgA, IgM, and IgG within the normal range were reported (29, 38, 47, 48). Moreover, Drozdowska et al. found no differences in IgG and IgM concentrations between 39 children of KTR and 39 age-matched controls (age one day-15 years) (48). At birth low numbers of total lymphocytes and specific lymphocyte subsets were reported in three studies, but in 28 of these 31 children normal lymphocyte counts were found at a maximum follow-up of 2 years (39, 44, 46). Ono et al. reported a significantly lower percentage of transitional B cells (CD19^+^CD10^+^) and a higher expression of CD154 on CD4^+^ T cells in children exposed to tacrolimus compared to children exposed to cyclosporine (*p* = 0.029 and *p* = 0.009 respectively) (44). Pilarski et al. reported that in an offspring group (*n* = 10, range 5 months-9 years) compared to a control group, cyclosporine-exposed children had significantly higher numbers of CD45RA + R0- T cells and azathioprine-exposed children had significantly higher numbers of CD45RA-R0+ T cells, suggesting that cyclosporine exposure delayed T cell development and azathioprine exposure accelerated T cell development (45). Moreover, children exposed to cyclosporine had a lower and to azathioprine a higher expression of CD29 T cells compared to the control group (45).

In summary, normal response to vaccination and no opportunistic infection were reported but, especially at young age, the results show some alterations in numbers of immune cells in the transplant offspring group and two studies indicate an increased risk of hospitalization for infection.

### Neurobehavioral and Cognitive Follow-Up

Sixteen articles conducted specific tests on neurobehavioral development or cognition (11, 13, 29–31, 33, 37, 45, 47, 50–56), of which three are case reports (29, 53, 54). The studies show that neurological development is similar to the general population. Five articles described intelligence quotient (IQ) scores (11, 29, 30, 50, 52). No significant differences regarding global intellectual performance were found when comparing the transplant offspring with the general population or matched control groups at infant, toddler, pre-school, and school age (11, 30, 50). However, Morales-Buenrostro et al. reported that visuospatial working memory might be affected in preschool children born after KTx (*p* = 0.007) (50). No significant differences in IQ scores were found between children only exposed to cyclosporine and children exposed to both cyclosporine and azathioprine (30). Subgroup analyses with mothers taking MMF prior to their awareness of being pregnant did not show statistical differences in full scale IQ (50). Kocisezwska-Najman et al. found no differences in the distribution of IQ between children born to LiTx and KTx recipients, though children of KTR had significantly higher percentages of preterm birth and LBW (risk factors for lower IQ) (57) compared to offspring of LiTx recipients (52). Devresse et al. reported that 8/21 (38%) children had a grade repetition, which is lower than their country’s general population (60%) (13).

In 296 children neurodevelopmental follow-up was performed without comparison to a control group (31, 33, 37, 47, 53–55). In 87% no developmental problems were reported and in 13% developmental delays, such as the need for educational support or neurological abnormalities such as cerebral palsy, slightly delayed psychomotor development, and intellectual disability, were reported.

### Kidney Function

Eight studies mentioned specific results on kidney function (12, 13, 26, 32, 33, 58–60). No abnormalities in kidney function were reported in 96% (243/252) of the assessed children. Al-Khader et al. reported no signs of glomerular or tubular defects and no hypertension or proteinuria in 41 children born from KTR at a mean follow-up of 52 months (12). In 95% of these pregnancies a calcineurin inhibitor (CNI, 73% cyclosporine, 22% tacrolimus) was used. Giudice et al. also reported no renal abnormalities in 12 children born from KTR at a mean follow-up of 2.6 years (32). In all these pregnancies cyclosporine was used during pregnancy. Willis et al. reported 4/40 (10%) children with urinary tract abnormalities on ultrasound: one ureteropelvic junction obstruction, one unilateral scar, and two unilateral renal dysplasia (two female siblings). These two female siblings also had abnormalities on urine analyses. 50% of the mothers used cyclosporine during pregnancy. The reported 10% is significantly more than the general population (2.9%, *p* = 0.036) (33). Dębska-Slizien et al. reported one child with symptoms of glomerulonephritis out of 22 children born to KTR (26). Borek-Dziecioł et al assessed kidney function parameters (urea, creatinine, potassium, and sodium concentrations) in 40 infants (newborns and children aged >1 year, age not specified) born to mothers with a KTx and 40 control infants matched to gestational age. They did not find any significant differences between the KTx and the control group, nor did they find any differences between the immunosuppressive regimens use by the mothers (58). Shaheen et al. analyzed basic kidney function parameters in the blood of offspring born to KTR as well (median age 39 months, range 6–72 months) and did not find problems in renal functioning or integrity (59). Devresse et al. analyzed questionnaires of 21 children born after KTx aged 7–25 years. None of the children reported taking any chronic medication and no one reported a history of chronic kidney disease, renal stones, gross hematuria, or pathological urine dipstick at school medicine (13). Czaplinska et al. assessed liver function (AST, ALT) and kidney function (creatinine and urea) in 51 infants born to mothers with a LiTx (newborns and children aged >1 year, age not specified). They did not find significant differences between the LiTx group and the control group matched to gestational age and time period of birth, except for significantly lower ALT levels in the LiTx group (60).

### Other Findings

One study with a relatively large sample size (*n* = 199 live births) reported significantly more cases of acute bronchitis, systemic lupus erythematosus, and hyperactivity disorders in the KTx offspring compared to the matched control group (*p* = 0.007, *p* = 0.025, *p* = 0.038 resp.) (61). The same study reported a significantly higher number of hospitalizations in the transplant offspring group compared to the control group (65.8% vs. 45.6%, *p* < 0.001), without specifying the reason for admission (61). One study focused on ophthalmological follow-up in children aged 1 week till >6 years (not further specified); no differences in pathological findings between the offspring of LiTx, KTx and the control group were found (62).

Bar et al. reported no significant difference in the rate of severe disability in the long-term; there were 8% (*n* = 3) in the transplant offspring group (two cases of cerebral palsy due to extreme prematurity and one deaf child, probably due to a cytomegalovirus infection) and 2.4% (*n* = 1) in the primary renal disease group (63). In one case report and one retrospective cohort study, two cases of hepatoblastoma at young age (2.5 years and 18 months) were reported: one child of a LiTx recipient and one child of a KTR (36, 43). One child born to a mother with a HTx had a serious, probably hereditary, cardiac insufficiency (64).

### Fathers With a History of a Transplantation

One article (11), described longer term follow-up of children born to fathers with a history of a SOT (11). Four children born to three fathers with a KTx were described. At follow-up (age 4 months, 10 months, 11 months and 2 years 7 months) no abnormalities were found on physical examination except for one child with sickle cell trait. The height percentiles were 25th, 25th, 55th, and 75th percentile and the weight percentiles were 10th, 45th, 75th, and 95th. On the Bayley Scales Mental Development Index the offspring scored within the range of normal (82, 83, 105, and 120).

## Discussion

To the best of our knowledge this is the first systematic review evaluating the available data regarding longer term (>1 year) outcomes of children of SOT patients. In general, we found that pregnancy after SOT appears to have reassuring longer term outcomes. Most children had normal physical and neurobehavioral development, despite frequent preterm birth and/or LBW.

The included studies reported high percentages of preterm birth and LBW in infants of mothers with a SOT. Precise numbers of preterm birth and LBW could not be calculated since some of the articles only mentioned mean or median gestational age and birth weight without giving the number of children fulfilling the definitions. In general, women who get pregnant after SOT are a selected population of patients who do well after transplantation. In this selected population, in line with our results on perinatal outcomes, previous research reported high preterm birth rates of 32% after LiTx (65) and 43% after KTx (3). Preterm birth and LBW are associated with poor growth in the first 2 years of life, as well as lower motor and cognitive scores compared to term infants (57). Interestingly, this is not in line with the data on offspring of mothers with SOT presented in this systematic review. Length and weight development was within the normal range in almost all children, including in children that were born preterm and/or with LBW, suggesting that the effects of transplantation and immunosuppressive medication on these outcomes are transient. A possible explanation for this difference is that the underlying mechanism leading to preterm birth and LBW is different in SOT recipients compared to the general population. Placentation is affected by the history of a SOT and especially by KTx, and the vascular remodeling in pregnancy is likely to be affected by immunosuppressive medication (66). Of the immunosuppressive medication, fetal exposure to CNIs especially is concerning, since approximately 70% of maternal tacrolimus and 37%–64% of maternal cyclosporine concentrations reach the fetus. Corticosteroids freely cross the placenta but 90% is metabolized to inactive forms in the placenta and azathioprine cannot be converted to its active form in the human fetal liver (6). However, the rate of obstetric complications such as preterm birth and LBW is similar in post-transplantation pregnancies on different immunosuppressive regimens, suggesting that immunosuppressive medication is not the only factor affecting the risk of complications (6).

The included studies show that results of neurological and cognitive assessment are similar to the general population. The results of our systematic review are in line with the TPRI that also suggests that cognitive and physical development of the children (>1,500 children) is comparable to the general population although their data is subject to reporting bias because of collection via voluntary patient questionnaires (1).

On immunological follow-up some abnormalities were seen. Low numbers of lymphocytes shortly after birth are reported in studies included in this review (39, 44, 46) and in other studies with a follow-up of <1 year (67, 68). However, lymphocyte numbers normalized at longer follow-up. Some differences in levels of subtypes of immune cells between immunosuppressive medications were observed. The relevance of these findings is arguable since no differences in immunological complications between the different immunosuppressive regimens were reported. Moreover, in none of the 1,664 children were opportunistic or chronic infections reported. Three studies reported a significantly higher number of hospitalizations in children born to transplanted mothers, including a higher number of antibiotic prescriptions in one study (44, 49, 61). A possible explanation for the increased rate of hospitalization in the transplant offspring is increased alertness to possible problems by their mothers and/or doctors. It is likely that the upbringing of children is influenced by the SOT of the mother. For example, maternal anxiety about her own and the child’s health may lead to increased care seeking behavior (11). Besides, some of the complications such as the reported kidney abnormalities may be due to hereditary risk and are not necessarily linked to the transplantation itself.

Several limitations of this systematic review must be acknowledged. A main limitation is the large proportion of case reports and retrospective studies that may be subjected to publication bias. However, of the included studies 16/53 had a control group (58% of the offspring, *n* = 960). Furthermore, most data presented here focused on childhood outcomes. Only five studies included offspring aged >18 years in their study group. Another limitation is that the majority of births (78%) presented here are after KTx. Therefore, it is difficult to draw conclusions about differences between the types of SOT. Future research should focus on the long-term follow-up of offspring born after SOT at multiple time points and preferably into adult age, since it could be hypothesized that *in utero* exposure to immunosuppressive medication could lead to vascular damage which in turn leads to organ damage later in life. It seems plausible that immunosuppressive medication, which has nephrotoxic side-effects in the transplant population, affects the development of the kidneys in the offspring. Fortunately, the existing studies described here are reassuring. However, the majority of the offspring were evaluated at a relatively young age. It would be possible that there are already small (non-significant) health problems in these children that become apparent at an older age. Future research should assess if problems at later age arise. This would be in line with findings in antenatal exposure to cyclosporine in rabbits whereby nephrological abnormalities and systemic hypertension occur, worsening with advanced age (69).

In conclusion, this systematic review shows that the majority of offspring of SOT patients are healthy and develop well. These findings are encouraging for patients considering pregnancy after SOT and should be discussed in preconception counseling. However, this systematic review also shows that existing information is scarce and predominantly limited to small studies with young children. Larger and longer prospective studies with long-term follow-up into adulthood of these children are necessary to optimize pregnancy counselling of SOT patients.

## Abbreviations

HTx, heart transplantation; IQ, intelligence quotient; JBI, Joanna Briggs Institute; KTR, kidney transplant recipient; KTx, kidney transplantation; LBW, low birth weight; LiTx, liver transplantation; LuTx, lung transplantation; MMF, mycophenolate mofetil; NOS, Newcastle Ottawa Scale; PRISMA, preferred reporting items for systematic review and meta-analyses; SOT, solid organ transplantation.
