# Supplementary material for: Follow-Up of Offspring Born to Parents With a Solid Organ Transplantation: A Systematic Review
Source: Transpl Int. 2022 Aug 5;35:10565. doi: 10.3389/ti.2022.10565 (PMC9389717; doi:10.3389/ti.2022.10565)
Supplement: Supplementary file 1 [file Table1.docx]

**Table S1 Reproducible search string**

Pubmed search string

("Organ Transplantation"[Mesh] OR "Transplant Recipients"[Mesh] OR heart transplant*[tiab] OR cardiac transplant*[tiab] OR lung transplant*[tiab] OR pulmonary transplant*[tiab] OR liver transplant*[tiab] OR hepatic transplant*[tiab] OR pancreas transplant*[tiab] OR pancreatic transplant*[tiab] OR kidney transplant*[tiab] OR renal transplant*[tiab] OR small bowel transplant*[tiab] OR intestinal transplant*[tiab])

AND

("Pregnancy"[Mesh] OR "Gravidity"[Mesh] OR "Pregnant Women"[Mesh] OR Pregnan*[tiab] OR gravidit*[tiab] OR conception*[tiab])

AND
("Epidemiologic Studies"[Mesh] OR "Prognosis"[Mesh:NoExp] OR cohort*[tiab] OR follow-up[tiab] OR followup[tiab] OR risk[tiab] OR associat*[ti] OR relat*[ti] OR prospective*[tiab] OR "Case Reports" [Publication Type] OR outcome[tiab])

AND
("Child"[Mesh] OR "Infant"[Mesh] OR "Adolescent"[Mesh] OR child*[tiab] OR infant*[tiab] OR adolescen*[tiab] OR pediatr*[tiab] OR paediatr*[tiab] OR youth[tiab] OR teen*[tiab] OR school*[tiab] OR kids[tiab] OR neonat*[tiab] OR offspring [tiab] OR "Infant, Newborn"[Mesh] OR newborn*[tiab] OR prematur*[tiab] OR preterm*[tiab])

NOT

("Review" [Publication Type] OR "Meta-Analysis as Topic"[Mesh] OR "Review Literature as Topic"[Mesh] OR systematic[sb] OR systematic review[ti] OR meta-anal*[ti])

Performed at 6-4-2022
Results: 1102

Embase search string

(‘organ transplantation'/exp OR 'graft recipient'/exp OR 'heart transplant*’:ab,ti OR 'cardiac transplant*':ab,ti OR 'lung transplant*':ab,ti OR 'pulmonary transplant*' OR 'liver transplant*':ab,ti OR 'hepatic transplant*':ab,ti OR 'pancreas transplant*':ab,ti OR 'pancreatic transplant*':ab,ti OR 'kidney transplant*':ab,ti OR 'renal transplant*':ab,ti OR ‘small bowel transplant*’:ab,ti OR ‘intestinal transplant*’:ab,ti)

AND

('pregnancy'/exp OR 'pregnant woman'/exp OR pregnan*:ab,ti OR gravidit*:ab,ti OR conception*:ab,ti)

AND

('cohort analysis'/exp OR 'case control study'/exp OR 'cross-sectional study'/exp OR 'case report'/exp OR 'prognosis'/exp/mj OR cohort*:ab,ti OR 'follow-up':ab,ti OR followup:ab,ti OR risk:ab,ti OR associat*:ti OR relat*:ti OR prospective*:ab,ti OR outcome:ab,ti)

AND

('child'/exp OR 'infant'/exp OR 'adolescent'/exp OR 'newborn'/exp OR child*:ab,ti OR infant*:ab,ti OR adolescen*:ab,ti OR pediatr*:ab,ti OR paediatr*:ab,ti OR youth:ab,ti OR teen*:ab,ti OR school*:ab,ti OR kids:ab,ti OR neonat*:ab,ti OR offspring:ab,ti OR newborn*:ab,ti OR prematur*:ab,ti OR preterm*:ab,ti)

NOT

('review'/exp OR 'meta analysis (topic)'/exp OR 'systematic review'/exp OR 'systematic review (topic)'/exp OR 'systematic review':ti OR 'meta-anal*':ti)

Performed at 6-4-2022
Results: 1742
