# Supplementary material for: Follow-Up of Offspring Born to Parents With a Solid Organ Transplantation: A Systematic Review
Source: Transpl Int. 2022 Aug 5;35:10565. doi: 10.3389/ti.2022.10565 (PMC9389717; doi:10.3389/ti.2022.10565)
Supplement: Supplementary file 2 [file Table2.docx]

**Table S2A, Summary of the included studies**, **cohort studies**

| **Author (Year),**  **country** | **Study type** | **Transplanted organ, number of children** | **Immunosuppressive regimen** | **Gestational age (weeks)** | **Birth weight (gram)** | **Outcome measures** | **Long-term outcome** | **Bias appraisal score** |
| --- | --- | --- | --- | --- | --- | --- | --- | --- |
| Devresse (2022), Belgium.^13^ | Retrospective cohort study. | Kidney: 43 infants (2 twins) from 32 women (57 pregnancies), 48% female. | Tac: 7 women,  Csa: 28 women,  MMF: 4 women,  Aza: 29 women,  Steroids: 36 women  Not described wat combination was used. | 37 (86%) preterm | 21 (46%) LBW | Median follow-up 17 years (range 7-25).   - Questionnaire send to 43 children or their parents if <18 years. 21 responded. Questions on current situation (weight, height, familial status and treatment), medical history (hypertension, diabetes and depression), addictions (smoking, etc) and educational. | - 2 children had a medical history including transient hypothyroidism and recurrent otitis. - 2 children had surgery, one for phimosis and one for bilateral uretro-vesical reflux procedure. - None of the children took chronic medication, including no hypotensive or diabetic treatment. - None of the children reported history of chronic kidney disease, hearing impairment, renal stone, gross hematuria or pathologic urine dipstick test screening at school medicine. - 8 children reported grade repetition and 2 were followed for attention deficit disorder during childhood. - On a 1-10 scale median self-estimation of physical and psychological health were 9.4 (range 7.5-10) and 9.5 (range 7-10). | Selection: ☆☆  Comparability: -  Exposure: ☆ |
| Egerup (2021), Denmark.^49^ | Nationwide controlled retrospective cohort study.  All children born to kidney-transplanted women between 1964  (when the first kidney transplantation was performed in Denmark)  and December 31, 2016 were identified.  end of the study period  (December 31, 2017).  Control group exact matched on gender, birth  year, and number of siblings at birth in a ratio of 1:10. No exact match in 9 infants. | Kidney: 124 infants.  Control: 1231 infants. | No data. | Mean 35.02 +- 3.23  Preterm: 57 (46.0%)  Control:  Mean 39.67 +- 3.23  Preterm: 50 (4.1%) | Mean 2421.6 +- 766.4  LBW: 47 (37.9%)  Control:  Mean 3444.1 +-569.1  LBW: 37 (3.0%) | Median follow-up 14.5 years (IQR 7.1-22.8).  Median follow-up of the control group 14.1 years (IQR 6.6-25.4). Subjects were included at birth and followed until the  time of an event, emigration, death, or the end of the study period.  Outcomes were defined based on administrative diagnosis codes identified in the Danish National Patient Register and the Danish Medical Birth Register relating preterm birth, malformation, LBW, and hospitalization due to infections. Study outcomes relating to redemptions of a prescription for antibiotic treat- ment were identified in the Danish National Prescription Register. Outcomes for both hospitalizations due to in- fections and redemption of a prescription for antibiotic treatment were evaluated overall (from birth to age 5 years), early (within the first year of life), and late (from age 1-5 years). | 11.3% (n=14) had a malformation compared to 5.7% (n=70) in the control group (risk ratio 1.98). 50% of the malformation in the KT group were cardiovascular malformations; of these the majority (6 cases) had malformations in relation to the septum (eg, ventricular septaldefect and atrial septal defect).   - No significant differences in infant mortality (death during the first year of life) were observed. - The average number of hospitalizations due to infection was higher in the KT group compared to the control (41.9% vs 24.8%, risk ratio 1.67 [1.33-2.09]). - During the first year of life 21% in the KT were registered with hospitalization due to infections compared to 10.8% in the control group (risk ratio 1.94 [1.33-2.82]). In the KT group, 5 cases had more than 1 hospitalization due to infection. The diagnosis   included bacterial sepsis in newborn (6 hospital contacts), unspecific virus infection (6 hospital contacts), laryngitis (4 hospital contacts), and respiratory infection (eg pneumonia and bronchitis) (6 hospital contacts). The median of the hospitalization time was 2 days (IQR 1-10), compared to 2 days (IQR 2-6) in the control group.   - From age 1-5 years 34.2% in the KT compared to 17.8% in the control were registered with hospitalization due to infection (risk ratio 1.89 [1.44-2.48]). In the KT nine cases had more than 1 hospitalization due to infection. The diagnosis included gastroenteritis (12 hospital contacts), pneumonia (8 hospital contacts), bronchitis (5 hospital contacts), acute obstructive laryngitis (8 hospital contacts), pharyngitis (5 hospital contacts), acute and chronic middle ear infection (6 and 8 hospital contacts), and urinary tract infection (eg,pyelonephritis and cystitis) (5 hospital contacts). The median of the hospitalization time was 2 days (IQR 1-3) in the KT and 2 days (IQR 2-3) in the control. - No significant differences   were observed regarding redemptions of prescription for antibiotic treatment from age 0-5 years (62.1% KT vs 57.4% control, risk ratio 1.08 [0.93-1.24]).   - The average number of antibiotic prescriptions filled between age 1 and 5 was increased in the KT (2.15; 95% CI, 1.65-2.65) compared with the control (1.49; 95% CI, 1.38-1.61). - The cumulative incidence of severe infections was higher in the KT and the difference between the 2 groups increased in relation to time. - In subgroup analyses, the outcomes of the preterm children were compared in the KT and the control group. Risk ratios for late hospitalizations due to infections (RR 2.02; 95% CI, 1.12-3.65) and the difference between average number pertaining to overall (0.61; 95% CI, 0.03-1.20) and late number of infections (0.61; 95% CI, 0.23- 0.98) continued to be significantly increased. - In the subgroup analysis with children born with LBW, the dif- ference regarding average number of hospitalizations due to late infections observed a borderline significant increase (0.43; 95% CI, −0.01-0.88). - No significant differences were observed in a subgroup analysis of children to mothers with diabetes. - Subgroup analyses comparing outcomes between different time periods (children born before 1996, from 1996 to 2010, and from 2010) results remained principally unchanged - No significant differences were observed in a subgroup analysis of children to mothers with hypertension. | Selection: ☆☆☆☆ Comparability: ☆☆ Exposure: ☆☆ |
| Borek-Dziecioł (2020), Poland.^58^ | Cross-sectional cohort study  Control group born at a similar gestational age. | Kidney: 40 infants  Control: 40 infants | CNI: 3 women,  CNI + GS: 16 women,  CNI + AZA + GS: 17 women,  AZA + GS: 2 women,  CNI + MMF: 1 woman,  Everolimus + AZA + GS: 1 woman.  MMF and everolimus were stopped immediately after the pregnancy was identified. | Not described. Kidney and control group born with similar gestational age. | Not described. | Newborns, infants, and children over 1 year of age were examined. Not described at what age.   - Renal parameters: urea, creatinine, potassium and sodium concentration were analyzed. - Urea serum concentrations were determined by the enzymatic method with urease and glutamate dehydrogenase. The upper limit for urea concentration is 48 mg/dL. - Creatinine was determined by the kinetic colorimetric method with alkaline pyruvate. Creatinine values were assumed to be within the normal ranges: 0.3-1.0 mg/dL for newborns, 0.2-0.4 mg/dL for infants, and 0.3-1.0 mg/dL for children >1 year of age. - Sodium and potassium serum concentrations were determined using indirect potentiometry. Sodium concentration was assumed to be within the normal range of 135-145 mmol/L and potassium 3.5-6.0 mmol/L for newborns and 3.5 to 5.5 mmol/L for infants and children. | - Elevated serum creatinine concentration was observed in 1 neonate from the KTx study group and in none of the children from the controls P=0.500). The mother of the newborn with abnormal creatinine concentration used CNI and GS. - The mean serum creatinine in the KTx group was 0.46 +-0.23 mg/dL and 0.47 +-0.20 mg/dL in controls (p=0.296). - Subgroup analyses of the mean serum creatinine concentration showed no significant differences between the different immunosuppressive regimens (CNI: 0.43 +-0.15 mg/dL, CNI + GS: 0.47 +-0.30 mg/dL, CNI + GS + AZA: 0.45 +-0.19 mg/dL, p=0.919). - Elevated urea serum concentration was not observed in the KTx group, and in 2 newborns of the control group (p=0.247). - The mean urea level in the KTx group was 24.3 +-9.71 mg/dL and in the control group 25.7 +-10.7 mg/dL (p=0.780). - Subgroup analyses of the different immunosuppressive regimens showed no significant differences in the urea levels (CNI: 26.0 +-3.61 mg/dL, CI + GS: 26.6 +-10.2 mg/dL, CNI + GS + AZA: 21.6 +-10.4 mg/dL, p=0.554) - Potassium levels in the KTx group ranged from 3.7-6.5, mean 4.77 +-0.59 mmol/L and in the control group from range 4.0-6.7, mean 5.0 +-0.71 mmol/L (p=0.189). - Hyperkalemia was observed in 10% (4/40) of children from the KTx group and in 20% (8/40) of children from the control group (p=0.348). - Mothers of children from the KTx group with hyperkalemia were prescribed different treatment regimens: 2 mother CNI + GS + AZA, 2 mothers: CNI + GS. - Subgroup analyses of the different immunosuppressive regimens showed no significant differences in the potassium concentrations (CNI: 4.67 +-0.21 mmol/L, CNI + GS: 4.67 +-0.69 mmol/L, CNI + AZA + GS: 4.89+-0.58 (p=0.205)). - Sodium levels in the KTx group ranged from 136-155, mean 142.3 +-4.62 mmol/L and in the control group range: 132-163, mean 144.3 +-6.59 mmol/L (p=0.140). - No hyponatremia was found in the study group; in the control group, mild asymptomatic hyponatremia (132-133 mmol/L) was observed in 2 children (5%); both were children over 1 year of age (P=0.247). - Hypernatremia was observed in 22.5% (9/40) of children from the KTx group (6 newborns and 3 children >1 year of age) and in 32.5% (13/40) children from the control group (6 newborns and 7 children >1 year of age) (P=0.317). - Mothers of children from the KTx group with hypernatremia were on different immunosuppressive regimens: 1 mother: CNI, 3 mothers: CNI + GS + AZA and 3 mothers: CNI + GS. - Subgroup analyses of the different immunosuppressive regimens showed no significant differences in the sodium concentrations (CNI: 144.0 +-9.17 mmol/L, CNI + GS: 142.1 +-4.95 mmol/L, CNI + GS + AZA: 142.6 +-3.77 mmol/L, p=0.851). - In the two children exposed to MMF or everolimus at the beginning of the pregnancy, the levels of all biochemical parameters analyzed were within the normal range. | Inclusion criteria: yes Study subjects: no Exposure measured: N.A. Measurements objective: yes Confounding factors: no Strategies against confounders: no Outcome measurements: yes Statistical analysis: yes |
| Dębska-Slizien (2020), Poland.^26^ | Retrospective cohort study. | Kidney: 25 infants  (31 pregnancies) in 26 women | 4 women: prednisone + CsA + AZA,  4 women: prednisone + CsA,  2 women: prednisone + AZA,  8 women: prednisone + TAC + AZA,  3 women: prednisone + TAC,  3 women: prednisone + TAC + MMF,  1 woman: prednisone + CsA + MMF,  1 woman: prednisone + TAC + sirolimus.  MMF was converted to AZA or discontinued in the first trimester.  Sirolimus was discontinued in the 4^th^ week of pregnancy. | Mean: 35 +-4 (median: 36, range: 24-40).  Preterm birth: 15 (60%) | Mean: 2363 +-1029 (median: 2400, range 490-4100)  LBW: 13 (52%),  VLBW: 6 (19%), ELBW: 4 (14%) | Median follow up 9 years (range 0.5-30 years).   - No specific long term outcomes described. | - 22 children were considered healthy at birth, 2 children died in the neonatal period and 2 children had congenital defects. 1 child a congenital neurological defect and 1 child was unhealthy at birth due to complications of prematurity. - 20 of the 22 children healthy at birth had good long term development. - 1 child was reported to have complications of prematurity at follow-up: retinopathy, hemiparesis requiring pediatric surveillance. - 1 child was observed due to symptoms of glomerulonephritis (also present in the mother before end-stage kidney disease). - The pregnancy with sirolimus exposure in the first trimester had a favorable outcome and no pediatric complications in the follow-up. - Of the 4 pregnancies with MMF exposure in the first trimester, 2 pregnancies ended with IUVD, 1 with a spontaneous abortion and 1 had a successful outcome with no reported abnormalities at follow-up. | Selection: ☆☆  Comparability: - Exposure: - |
| Bachmann (2019), Germany.  ^40^ | Two-center retrospective cohort study | Kidney: 30 infants  Combined kidney-pancreas: 2 infants  (28 women)  2 women had a kidney-pancreas transplantation. Unclear how many children they had. | 20 women: TAC + prednisolone,  6 women: CsA + prednisolone,  2 women: CsA + AZA + prednisolone,  2 women: TAC,  1 women: AZA,  1 women: TAC + AZA + prednisolone. | Median: 34.7 (IQR: 31.9-36.3)  Extremely preterm: 4 (12.5%)  Very preterm: 4 (12.5%)  Preterm: 17 (53.1%) | Median: 2030 (IQR: 1380-2375)  LBW: 25 (75.7%) | Evaluation of anthropometric measures at birth, 12 and 24 months. 65.6% of the children had a complete dataset at 24 months. Preterm and term pregnancies were compared.  Physical and psychomotor development examination by a pediatrician, collected from the patient file:  At birth, 12 months and 24 months (+- 2 months): weight, length and head circumference.   - In order to achieve age-related variables, corrected age based on expected date of birth in preterm deliveries was used. - Weight was measured with an electronic scale accurate to the nearest 1 g. - Length was measured with an infant-length board to the nearest 1 mm when the weight was >1000 g. or with a nonstrech measuring tape for very preterm infants in a closed incubator. - Head circumference was measured in the largest frontoocipital plane to the nearest 1 mm with a nonstretch measuring tape - Weight, length and head circumference were converted to the age-specific and gender-specific Z-scores according to the WHO growth chart.   Questionnaire filled in by the mother about the physical developmental status of the child containing:   - physical examination, - anthropometric measures, - medical and paramedical history. | - At 12 and 24 months all children but 1 had adequate weight and height for age. 1 child was small for age. - At birth the weight, length and head circumference of the preterm group was significantly lower than the term group (p=0.002, p=0.006, p=0.011 resp.). - At 12 months no significant differences between the preterm and term group with regard to weight, length and head circumference were visible (p=0.704, p=0.71, p=0.365 resp.). - At 24 months no significant differences between the preterm and term group with regard to weight, length and head circumference were visible (p=0.769, p=0.308, p=0.410 resp.). - At birth the weight-for-age, length-for-age and head-circumference-for-age Z-scores were resp. -1.04 (IQR -1.43, -0.54), -0.79 (IQR: -1.43, -0.15) and -0,65 (IQR: -1.4, 0.0075). - At 12 months the weight-for-age, length-for-age and head-circumference-for-age Z-scores were resp. -0.375 (IQR: -0.82, 0.1), -0.07 (IQR: -0.69, 0.028) and -0.28 (IQR: -0.74, 0.51). - At 24 months the weight-for-age, length-for-age and head-circumference-for-age Z-scores were resp. -0.31 (IQR: -0.94, 0.32), -0.78 (IQR: -1.15, -0.17) and -0.05 (IQR: -1.1, 0.36). - The following abnormalities were observed at birth: 1 child with: microcephaly, macrosomia and cryptorchism and 1 child with strabism and bronchial asthma. - The physical examination was normal in 30/32 infants (93.75%) at 12 months and 20/21 (95.2% at 24 months. - In 2 very preterm children mild neurological deviations at 12 months were found: muscle tone disturbances in lower limbs in 1 child and slightly reduced muscle tone in the other child. At 24 months follow-upthe deficiencies had improved. | Selection: ☆☆☆ Comparability: ☆ Exposure: ☆☆ |
| Morales-Buenrostro (2019), Mexico.^50^ | Multicenter cross-sectional study.  Control group matched to socioeconomic background and gestational age. | Kidney:  50 infants (22 girls) from 47 women.  Control: 50 infants (18 girls) from 45 women. | All women: prednisone.  33 women: AZA.  36 women: CsA.  7 women: TAC.   23% of women (n=11) received MMF treatment prior to their awareness of being pregnant. In all cases, this treatment was discontinued as soon as they found out about their pregnancy. | Mean: 33.78 +/- 3.79  Control: 35.1 +/- 3.01  p = 0.06 | Mean: 2494.36 +/- 665.17  Control: 2750.16 +/- 769.94  p = 0.08 | Children aged 4 years and older were included. Most children were aged between 6-16 years at time of testing (n= 32 in the study group and n= 37 in the control group).   - Age-specific tests conducted at 1 time-point. - Interview with the mother and the child.   Intellectual performance tested with:   - 4-6-year-old children: WPPSI to calculate the verbal, performance and full scale IQ. - 6-16 year old children: WISC-IV to calculate the full scale IQ and four index scores: verbal comprehension, perceptual reasoning, working memory and processing speed. - 16 years and older: WAIS-III to calculate full scale IQ, verbal IQ and performance IQ. | - Global intellectual performance normal. - No significant differences in full-scale IQ score: mean IQ 95.65 (SD 15.36) against 94.06 (SD 12.96) in the control group (p = 0.89). - No significant differences in the subscales except for the time to complete the "Zoo locations" subscale of the WPPSI whereby the exposed group was significantly slower (179.78 (SD 56.2) seconds against 99.07 (SD = 25.91) seconds (p = 0.007). - Indicating that visuospatial working memory may be affected in preschool children. - Subgroup analyses with the mothers taking MMF prior to their awareness of being pregnant did not show statistical differences in full scale IQ. - Socioeconomic status was the only significant predictor for full IQ score in multivariate logistic regression. Immunosuppressive medication exposure was not a significant predictor. | Inclusion criteria: yes Study subjects: yes Exposure measured: N.A. Measurements objective: yes Confounding factors: yes Strategies against confounders: yes Outcome measurements: yes Statistical analysis: yes |
| Schreiber-Zamora (2019), Poland (1).^51^ | Single-center prospective cohort study.  Control group matched to gestational age and time period of birth. | Kidney:  36 infants.  Control: 36 infants. | 12 women: AZA + CsA + glucocorticoid  8 women: TAC + glucocorticoid  7 women: CsA + glucocorticoid  6 women: AZA + TAC + glucocorticoid.   In one case of unplanned pregnancy, MMF was used in the first 8 weeks of pregnancy. | Median both groups combined: 36 (range 27-40) (p=0.281)  Study group:  4 (11%) extremely preterm  Control group:  4 (11%) extremely preterm | Mean: 2318  Control group: 2613  p = 0.083 | Age-specific neurological examination conducted at 1 time-point. Follow up at median 3.12 years.  Re-evaluation after 3 years if abnormalities were found.   - Infants: psychomotor evaluation consisted of: locomotion, posture control, visual and motor coordination. Circumference and shape of the head as well as the size of the fontanelle and the peripheral cranial nerve were also assessed. - Early childhood: neurological examination included head circumference, head shape assessment and cranial nerve evaluation. Upper and lower limb strength, reflexes muscular tension and sensation were also tested.   Evaluation of neurological development was also done with ultrasound.  Abnormalities categorized into:   - Mild: slightly decreased or reduced limb muscle tension that did not affect the spontaneous activity, abnormal patterns of posture with a tendency for asymmetry with proper limb activity or with a tendency for deflection, small motor disorders in the form of delayed maturation of hand coordination or major motor disorders in the form of delayed maturation of equivalent reactions. - Moderate: increased or decreased muscle tone of limbs without significant effect on the spontaneous and spastic motion, axial tension disturbance in the form of increased spinal muscle tension, coexistence of small and large motor disorders manifested by deterioration in motor coordination. - Severe: cerebral palsy, persistent movement and postural impairments that limited activity.   Hypotrophy was diagnosed with UK-WHO growth charts to determine the percentile of birth weight. The rank was determined based on gestational age and sex of the infant. | - Normal neurological development in the majority of the children: 86% in both the study group and the control group (p = 0.999). - Mild deviations in 11% (n= 4) in the study group against 14% (n=5) in the control group (p = 0.999). - 1 case of moderate deviations was found in the study group. - No cases of severe deviations in both groups. - Almost all (8/10) children with neurological deviations were born preterm in good general conditions. - Ultrasound examination showed in 4/5 cases with neurological deviations no abnormalities. In 1 case a second grade intraventricular hemorrhage was reported. - The neurological deviations observed in the first year of life were mild and transient. - 6 children in the study group had hypotrophy. 2 of them had mild and 1 moderate deviations in the neurological examination. - No differences in the frequency of neurological deviations in children born with hypotrophy between the study group and the control group (study group 8%, control group 3%, p=0.305). | Selection: ☆☆☆☆  Comparability: ☆ Exposure: ☆ |
| Schreiber-Zamora (2019), Poland (2).^42^ | Single center prospective cohort study  Control group matched to gestational age and time of birth. | Liver: 35 infants  Kidney: 26 infants  Control: 64 infants | 35 women: TAC  55 women: glucocorticoids  4 women: TAC,  31 women: TAC + another not specified immunosuppressive drug  55 women: glucocorticoids + another not specified immunosuppressive drug | Mean Tx: 36.3 +-2.4  Control: 36.9 +- 2.9  p=0.999 | Mean Tx: 2654 +- 655  Control: mean: 2968 +- 770  Tx vs control p=0.209  Liver: mean: 2888 +- 611 (range: 1420-4100)  LBW: 8 (23%)  Kidney: mean: 2340 +-601 (range: 580-3450)  LBW: 13 (50%)  ELBW: 2 (8%)    Control matched to liver: mean: 3115 +-717 (range 1315-4720)  Control matched to kidney: mean: 2754 +- 810 (range: 920-4220) | Measurement of BMI was performed as a one-time measurement on one of the follow-up visits.  Age at BMI measurement in the Tx group:   - 6 children 1-12 months old - 15 children 1-3 years old - 25 children 3-6 years old - 15 children >6 years   Age at BMI measurement in the control group:   - 7 children 1-12 months old - 16 children 1-3 years old - 24 3-6 years old - 17 >6 years - Up to 5 years of age BMI-for-age growth charts for boys and girls aged 0-24 months and 24-60 months of life were used. - In children aged >5 years of age the BMI-for-age 5-18 years for boys and girls was used.   The analyzed population was divided into subgroups in relation to BMI values:   - Underweight: BMI < 5^th^ centile - Normal weight: BMI 5-85^th^ centile - Overweight: BMI 85-95^th^ centile - Obesity: BMI ≥95^th^ centile | - No differences in the prevalence of underweight and overweight when comparing the study group to the control group and to the general population. - No difference when comparing the prevalence of underweight in LiTx (5.7%) and Ktx group (3.8%) to the LiTx control (5.3%) and KTx control (7.7%) (p=0.94, p=0.55 resp.) - No differences when comparing the prevalence of overweight in LiTx (5.7%) and Ktx group (11.5%) to the LiTx control (5.3%) and KTx control (7.7%) (p=0.94, p=0.64 resp.). - There was a trend towards obesity in the children of the KTx group compared to the control group (16 vs 6%, p=0.072). - The theoretical incidence of obesity of the general population (5%) was significantly lower than the incidence in the LiTx (17.1%) and KTx (15.4%) a p<0.001 and p=0.02 resp. - Among the children diagnosed with obesity no case of fetal growth restriction was reported. - Intrauterine hypertrophy was found in 3 obese children in the Tx group and in 0 children of the control group. - Prenatal exposure to TAC was associated with an 2.8 fold increase for developing a higher BMI in later follow-up. | Selection: ☆☆☆☆ Comparability: ☆ Exposure: ☆☆ |
| Turkyilmaz (2018),  Turkey.^14^ | Retrospective single center cohort study. | Liver: 8 infants. | 8 women: TAC. | Mean: 37.2 +/- 2.1 (range 34 - 40).  3 (37.5%) preterm | Mean: 2852 +/- 562 (range: 2150-3470) | Mean follow up 3.2 years +/- 2.4 years, range 1-7 years.  Retrospective analyses of patient records.   - No specific long term outcome measurements described. | - All of the children were healthy at last follow up. - 1 child had renal pelvis dilatation which resolved spontaneously. | Selection: ☆☆☆ Comparability: - Exposure: ☆ |
| Kociszewska-Najman (2018),  Poland.^52^ | Single-center prospective cohort study.  Control group matched to gestational age and time of childbirth. | 78 infants: Liver: 42 infants.  Kidney: 38 infants.  Control: 78 infants. | 40 women: TAC,  20 women CsA,  2 women: AZA,  11 women: AZA + CsA,  5 women: AZA + TAC,  66 women steroids. | Mean: 37.0 +/- 2.6  Control:  37.0 +/- 2.8  p > 0.999 | Mean: 2653 +/- 655  Control:  2880 +/- 791  p = 0.053 | Comparison of intelligence levels with 3 age-appropriate psychological tests. 1 assessment per child (n= 31 < 30 months, n= 47 >30 months).  Psychological examination performed by qualified clinical psychologists to evaluate mental development: speech, thinking, verbal comprehension, grasping manipulation, memory, visual processing and grapho-motor skills. Results expressed in IQ.   - Up to 30 months: Psyche Cattell Infant Intelligence Scale, - 2-7 years: Terman-Merril Intelligence Scale, - 5-11 years: Scales of Raven’s Progressive Matrices. | - No significant differences in the distribution of the IQ between KTx and LiTx children nor with the control at infant, toddler, pre-school and school age children. - In infants and toddlers no differences in the Psyche Cattell Infant Intelligence test were found between the Tx group and the control group (p = 0.537). - In preschool children the average IQ was more often diagnosed in the Tx group (36% vs 23%, p = 0.321). In the control group the high and very high IQ were more often diagnosed (32% vs 45%, p = 0.353). - In school age children no differences between the Tx group and the control group were observed. | Selection: ☆☆☆☆ Comparability: ☆ Exposure: ☆☆ |
| Ono (2015),  Brazil.^44^ | Prospective cohort study.  2 control groups: not matched to gestational age and a matched to gestational age group. | Kidney: 28 infants (13 girls, 1 twin).  Control group 1: 40 infants.  Control group 2: 28 infants. | All women: AZA (5-150 mg/day) + prednisone (5 mg).  19 women: TAC (2-8 mg/day).  8 women: CsA (5-250 mg/day).  1 woman: MMF (until pregnancy diagnostic). | Median: 37.2 (range 31.1 - 41.0).  14 (50%) preterm  Control group 1: 39.6 (range 37.4-41.3)  0 preterm  Control group 2: 37.0  Tx group vs Control group 1  p < 0.001  Tx group vs Control group 2: p= 0.786 | Median: 2607 (range: 1219-3685).  Control group 1: 3312.5 (range 2785-3755)  Control group 2: 2805.0  Tx group vs Control group 1:  p < 0.001  Tx group vs Control group 2: p= 0.770 | Immunological follow up and general follow up all compared to control group 1. Immunological follow up (blood sample) at birth and at 8 months of age. General follow up by the pediatrician every month during the first 6 months, every 3 months until 2 years of age.   - Blood sample collection at birth from the umbilical cord and at 8 months from a peripheral vein. - Immuno-phenotypic studies were done with fresh blood. Each sample was stained with fluorochromeconjugated monoclonal antibodies. - The following cells were identified:   naïve, central memory, effector memory and effector cells of CD4+, CD8+ T lymphocytes. Activated CD4+ and CD8+ T lymphocytes. Transitional, active memory, exhausted memory, resting memory and naïve B lymphocytes. NK cells, Gamma-Delta T cell, NKT cells and regulatory T cells.   - Expression of TLR2 on monocytes and granulocytes was also stained for. - In vitro cell activation of T cells and dendritic cells.   Tx group compared to control group 2 for:   - Factors associated with hospital admission were analyzed by univariate logistic regression. | - At birth: 16/20 (80%) of the children in the Tx group had low numbers of B cells. 3/20 (15%) had low CD8+ T cells and 5/20 (25%) had low NK cells.   At birth: the Tx group had significantly:   - lower numbers of platelets, white blood cells, neutrophils and eosinophils, - lower numbers of CD4+ T cells, B cells, NKT cells and activated CD8+T cells, - higher percentage of exhausted memory and activated memory B cells.   At 8 months: the Tx group had significantly:   - higher numbers of B cells, - lower percentage of exhausted memory B cells, CD4+, CD25+ and FoxP3+ cells, - Higher expression of CD103+ on CD4+ Treg cells.   4 children had CD4+, CD8+, B or NK cell numbers below the 10^th^ percentile at 8 months.   - 3 children reached normal values for age at 17.0, 18.4 and 19.4 months. - 1 child maintained low numbers of CD8+ T cells and borderline B cell numbers at 35.3 months. Despite these low numbers this child was never hospitalized or had recurrent infections during the entire study.   Expression of TLR2 on monocytes and granulocytes was similar in both groups.  TNF-a expression in plasmacytoid dendritic cells after in vitro stimulation was lower at birth in the Tx group but similar at 8 months.  All other parameters were similar between the groups at birth and at 8 months.  At 12 month follow up:   - All children survived. - 8 (28.6%) from the Tx group and 3 (7.5%) from control group 1 were hospitalized due to infectious disease. - All children that were hospitalized were exposed to TAC during pregnancy. - Children exposed to TAC compared to CsA had significantly lower percentage of transitional B cells (p= 0.029) and significantly higher expression of CD154 on CD4+ T cells after stimulation (p=0.009).   Logistic regression comparing the Tx group with control group 2:   - Children from the Tx group had 4.351 times higher chance of hospital admission than control (p = 0.046). | Selection: ☆☆☆☆ Comparability: ☆ Exposure: ☆☆ |
| Czaplinska (2014), Poland.^60^ | Cross-sectional cohort study.  Control group: similar gestational age, born in the same time period. | Liver: 51 infants.  Control: 51 infants | CsA: 4 women,  CsA + prednisone: 7 women,  TAC: 8 women,  TAC + prednisone: 26 women,  CsA + AZA + prednisone: 1 woman, TAC + AZA + prednisone: 5 women. | Not described.LiverTx and control group born with similar gestational age. | Not described. | Neonates, infants, and children >1 year of age were examined. Not described at what age.   - Analysis of liver parameters: alanine transaminase (ALT) and aspartate transaminase (AST). Normal level of ALT: <40 U/L for girls and <50 U/L for boys. Accepted AST norms were <140 U/L for newborns and <55 U/L for children and infants older than 1 month. - Analysis of two kidney parameters (urea and creatinine). Normal creatinine levels were considered to be 0.3-1.0 mg/dL for newborns, 0.2-0.4 mg/dL for infants, and 0.3-1.0 mg/ dL for children older than 1 year. The upper limit of urine concentration was considered to be 48 mg/dL. | - In the LT group all children had normal levels of ALT. In the control group, 5 (9.8%) children had elevated levels of ALT (all <12 months of age). The average ALT level for the LT group was significantly lower that the control group (15.14 U/L, 22.6 U/L, p=0.0127). - 3 (5.9%) children from the LT group (TAC monotherapy or TAC + prednisone) and 8/51 (15.7%) children of the control group had increased AST levels. Incorrect AST levels were reported in all age groups, two newborns, four infants, and two children older than 12 months. The mean AST was not significantly different (LT: 44.8 U/L, control: 50.4 U/L, p=0.2003). - The abdominal sonographic scans were normal for all children with abnormal liver enzymes. - In the LT group 3 (5.9%) children had elevated and 3 (5.9%) had boundary high (1.0 mg/dL) levels of creatinine (all newborns), and in the control group one newborn had an elevated creatinine level (p=0.6175). None of these children had abnormalities in sonographic scans of the urinary system. The average creatinine level were not significantly different ((LT: 0.51 mg/dL, control: 0.44 mg/dL, p=0.224). - One newborn from the LT group had an elevated urea level and in the control group all children had normal urea concentrations. The mean urea concetrations were not significantly different (LT: 26.2 mg/dL, LT newborns: 27.3 mg/dL, control: 24.2 mg/dL, control newborns: 19.6 mg/dL. - The highest creatinine and urea levels were observed in newborns whose mothers had been administered TAC + AZA + prednisone or CsA + prednisone or CsA. - For older children, creatinine and urea levels were similar to the control group, unrelated to immunosuppressants administered to the mother. | Inclusion criteria: yes Study subjects: no Exposure measured: N.A. Measurements objective: yes Confounding factors: no Strategies against confounders: no Outcome measurements: yes Statistical analysis: yes |
| Norrman (2014),  Sweden.^61^ | Population based retrospective cohort study.  4 study groups:   1. Tx + IVF 2. Tx + no IVF 3. No Tx + IVF 4. No Tx + no IVF   Group 1 is matched to group 3 to maternal age, parity and date of birth.  Group 2 is matched to group 4 to maternal age, parity and date of birth. | Kidney:  Group 1: 7 infants (1 twin).  Group 2: 199 infants.  Control:  Group 3: 665 infants.  Group 4: 3980 infants. | Group 1: 4 women CsA,  2 women: CsA + prednisolone,  1 woman: prednisolone.  Group 2: medication unknown. | Group 1: mean: 37.1 +/- 4.5 (range: 27.3 - 40.4), 2 (28.6%) preterm.  Group 2: Mean 35.9 +/- 3.9 (range: 22.9-42.6)  96 (48.5%) preterm  Group 3:  Mean 39.5 +/- 2.3 (range 25.0-43.0)  59 (8.9%) preterm  Group 4: 39.8 +/- 1.9 (range 23.7-44.0)  206 (5.2%) preterm  Group 2 vs. 4: p <0.001 | Group 1: mean: 2795 +/- 1029 (range: 672-3640). 1 LBW, 1 VLWB, 1 SGA.  Group 2: mean 2522 +/- 850 (range: 415-4460). 43.7% (n=87) LBW, 11.6% (n=23) VLBW. 21.2% (n=42) SGA.  Group 3:  3441 +/- 624 (range 640-5460).  43 (6.5%) LBW  Group 4:  3505 +/- 551 (range 570-5700).  134 (3.4%) LBW  Group 2 vs 4: p <0.001 | Retrospective analyses of 5 registries: National Quality Register of Assisted Reproduction, the National Register in IVF, the Swedish Medical Birth Register, the National Patient Register and the Swedish Cause of Death Register.  Follow up  Group 1: mean age at follow up: 9.7 +/- 4.2 years. Group 2: mean age of follow up: 14.7 +/- 9.4 years.   - No statistical comparison between group 1 and the control group due to low numbers. | Group 1:   - 2 children (29%) diagnosed with hyperactivity disorders. - 2 children (29%) had been hospitalized due to infectious disease.   Group 2:   - Significantly higher number of children hospitalized in the Tx group: 131 (65%) vs 1815 (45.6%) in the control group (p<0.001). - Acute bronchitis, systemic lupus erythematosus and hyperactivity disorders were significantly more common in the Tx group (p=0.007, p= 0.025, p=0.038 resp.). - Mortality after 1 year was similar in the Tx group compared to the control group (p=1.00). - No other significant differences in the long term outcomes were found between the Tx group and the control group. | Selection: ☆☆☆☆ Comparability: ☆ Exposure: ☆☆ |
| Drozdowska-Szymczak (2014),  Poland.^48^ | Single center prospective study.  Control group matched to similar period of birth and similar gestational age. | Kidney: 39 infants.  Control: 39 infants. | 3 women: TAC, 7 women: TAC + glucocorticoids,  9 women: CsA + glucocorticoids,  2 women: AZA + glucocorticoids,  7 women: AZA + TAC + glucocorticoids,  10 women: AZA + CsA + glucocorticoids,  1 woman: TAC + MMF (pregnancy diagnosed at the beginning of the second trimester). | Range 27-38.  n= 12 preterm  Control:  16 preterm | Range 580-3160.  n= 20 LBW  Control:  n= 19 LBW | Immunological follow up.  Serum IgG and IgM measurements at 1 time-point. Follow up range: 1 day-15 years (n= 26 was 10 months or older) and in the control group 1 day till 14 years.   - Serum IgG and IgM was evaluated with agglutination immunoassays. | - Normal IgG concentrations were found in 82.05% (n=32) in the Tx group compared to 79.49% (n=31) in the control group (p=0.775). - IgG concentrations below the normal range were observed in 12.82% (n=5) in the Tx group compared to 15.38 (n=6) in the control group (p=0.746). - Normal IgM concentration were found in 53.85% (n=21) in the Tx group compared to 61.54% (n=24) in the control group (p=0.494). - IgM levels below the normal range were found in 38.46% (n=15) in the Tx group compared to 35.9% (n=14) in the control group (p=0.815). - Decreased levels of IgM and IgG were found in preterm and SGA infants in both groups. - In the Tx group low levels of IgG were found in 5 children, 2 below 2 months of age and 3 aged 5-10 years. - In the Tx group low levels of IgM were found in 15 children, 14 aged below 15 months and 1 aged 5-10 years. - In the children exposed to TAC 11.1% (n=2) had decreased levels of IgG and 50% (n=9) had decreased levels of IgM. - In the children exposed to CsA 10.5% (n=2) had decreased levels of IgG and 26.3% (n=5) decreased levels of IgM. - In the children exposed to AZA 26.3% (n=5) had decreased IgG levels and 47.4% (n=9) decreased IgM levels. | Selection: ☆☆☆☆ Comparability: ☆ Exposure: ☆ |
| Kociszewska-Najman (2013),  Poland.^62^ | Prospective cohort study with control group. | 82 infants: Liver:  37 infants.  Kidney: 45 infants.  Control: 66 infants. | LiTx:  6 women: TAC,  3 women: CsA,  20 women: TAC + glucocorticoids,  7 women: CsA + glucocorticoids,  1 woman: AZA + CsA + glucocorticoids.  KTx:  2 women TAC,  8 women: TAC + glucocorticoids,  9 women: CsA + glucocorticoids,  1 woman AZA + glucocorticoids,  5 women: AZA + TAC + glucocorticoids,  10 women: AZA + CsA + glucocorticoids | LiTx: median: 37 (range: 33 - 41), 43.2% (n=16) preterm  KTx: median: 36 (range: 27-39) 68.6% (n=24) preterm  Control: median: 37 (range 27-41)  LiTx vs KTx: p = 0.03052  Tx group vs control: no p mentioned. | LiTx: median 2930 (range: 1420 - 4100).  KTx: median 2530 (range 580 - 3450).  Control: median: 3065 (range: 1190-4720).  LiTx vs KTx: p = 0.000923.  Tx group vs control: p = 0.006334. | Retrospective analyses of the parameters in the neonatal period of the child.  Prospective ophthalmological examinations by a pediatric ophthalmologist at 1 of the following moments: neonatal (1-4 weeks of age), babyhood (2-12 months), early kindergarten (1-3 years), later kindergarten (4-6 years) and school years (>6 years). Not all children at all follow up moments tested. Most children tested in the late kindergarten stage.   - Neonatal stage (1-4 weeks): mainly done in preterm infants in danger to develop retinopathy of prematurity. | No significant difference in percentage of pathological findings in ophthalmological examinations was found:   - between the LiTx and KTx group. (LiTx group: 16.2%, KTx group 17.1 %, p=1.00), - between the Tx group (LiTx and KTx) compared to the control group (23.6%, p=0.6206).   The disorders detected in the LiTx and KTx groups as well as the control group were similar.   - In the LiTx group convergence insufficiency was diagnosed in 5 children and exotropia in 1 child. - In the KTx group Retinopathy of prematurity was diagnosed in 2 children, hyperopia in 2 children, exotropia in 1 child and convergence insufficiency in 1 child. - In the control group exotropia was diagnosed in 4 children, convergent strabismus in 3 children and convergence insufficiency in 2 children. | Selection: ☆☆☆ Comparability: - Exposure: ☆ |
| Shaner 2012,  United States.^16^ | Retrospective multicenter cohort study. | Lung:  18 infants in 21 women (30 pregnancies) (1 triplet). | 6 women: CsA + AZA + prednisone,  1 woman: CsA + AZA, 1 woman: CsA + prednisone,  2 women: CsA + AZA + predisone,  12 women: TAC + AZA + prednisone,  3 women: TAC + AZA,  3 women: TAC + prednisone,  1 woman: TAC,  1 woman: TAC + MMF + prednisone. | Mean 33.9 +/- 5.2.  11 preterm | Mean 2206 +/- 936.  11 LBW, 3 VLBW | NTPR registry and retrospective questionnaires.   - No specific long term outcomes described.   Follow up mean: 7.0 years (+/- 5.37), range: 1.25 till 17.36 years. | - At last follow-up, all 16 surviving children were reported healthy and developing well. - 2 infants died in the neonatal period. | Selection: ☆☆☆  Comparability: - Exposure: ☆ |
| Nulman (2010),  Canada.^30^ | Prospective multicenter cohort study.  Control group not exposed to CsA but used other non-teratogens. Matched to maternal age at conception, alcohol and tobacco use, child’s age and sex. | Kidney: 39 infants in 26 women.  Control: 38 infants in 38 women. | All women: CsA,  18 women: CsA + AZA,  25 women: prednisone.  (AZA dose: 39.84 +/-43.09, CsA dose: 208.59 +/- 108.25, prednisone dose: 7.76 +/- 3.21) | Mean: 36.77 +/- 2.88.  n= 13 (33%) preterm:  4 at 36, 5 at 35, 1 at 32, 2 at 30, 1 at 27  Control:  Mean: 39.48 +/- 1.75   p<0.001 | Mean: 2650.32 +/- 69912.  7 infants IUGR  Control: 3487.40 +/- 593.86  P<0.001 | Mean follow up 8.06 years, range: 3 years 7 months till 15 years 9 months.  Primary outcome: full scale IQ, verbal IQ and performance IQ.  Retrospective questionnaires about immunosuppressive drug use, lifestyle, comorbidities and the pregnancy.  Prospectively collected:  Physical examination of the child:   - weight, length and head circumference.   Psychological examination of mother and child conducted by a trained psychologic assistant under supervision of a registered psychologist.   - Blinded assessment. - Standardized age-appropriate validated neuropsychologic tests were used. - Full scale IQ, verbal IQ and performance IQ were assessed with the WPPSI-R. - Visuomotor abilities were assessed with the VMI-4 and the WRAVMA. - Mothers completed the CBCL to assess psychologic adjustment.   The intelligence, presence of depression and the SES of the mother were assessed as potential confounders.   - The mother’s full scale IQ was determined with the Vocabulary and Matrix Reasoning subtests of the Wechsler Abbreviated Scale of Intelligence (WASI). - Depression was assessed with the Center for Epidemiologic studies Depression Scale (CES-D). - SES was assessed with the Hollingshead Four Factor Index of Social Status. | - At follow up there was no significant difference in the weight, height and head circumference of the Tx group compared to the control group. - No statistical differences in the full scale IQ, verbal IQ and performance IQ between the Tx group and the control group. - No statistical differences in the full scale IQ, verbal IQ and performance IQ between children exposed to CsA only and children exposed to both CsA and AZA. - Preterm children exposed to CsA compared to full term children exposed to CsA had significantly lower full scale IQ and verbal IQ scores but no differences in performance IQ. - No statistical children between preterm children with and without IUGR in full scale IQ, verbal IQ or performance IQ (p = 0.53, p = 0.25, p= 0.79 resp.). - Maternal IQ and SES were significantly positively associated to the child’s full scale IQ and verbal IQ. - No differences were found in behavior or temperament scores. - No significant differences in full scale IQ, verbal IQ or performance IQ between CsA exposed children who were breastfed and who were not. - In the Tx group no differences were found between children from single deliveries compared to children from multiple deliveries in cognitive outcome. | Selection: ☆☆☆☆ Comparability: ☆ Exposure: ☆☆ |
| Al-Khader (2004),  Saudi-Arabia.^12^ | Multicenter retrospective cohort study. | Kidney:  110 infants (65.4% girls) in 72 women (3 twins).  Follow up of 41 infants. | 73% CsA, 21.8% TAC, 5% rapamycin. 23% MMF. 22% AZA. 100% prednisolone. | 64% preterm | 84% under the 50th percentile, 19% under the 10th percentile | Renal follow up of 41 infants, mean follow up: 52 months (range: 13-83 months).  Retrospective analyses of medical records.   - No details on the method of follow up mentioned.   The following parameters were analyzed in 44 infants:   - Plasma urea, plasma chloride, plasma bicarbonate, plasma potassium, plasma phosphate, plasma creatinine.   In 14 infants the following was analyzed:   - urinary amino acids, - urinary RBC morphology, - urinary protein by dipstick. | - No glomerular or tubular defects, - No hypertension or proteinuria, - urinary amino acids and urinary RBC morphology normal. Urinary protein by dipstick negative. - 4 infants had a birth defect: 1 bilateral inguinal hernia, 1 umbilical hernia, 1 strawberry hemangioma, 1 cleft palate. | Selection: ☆☆☆ Comparability: - Exposure: ☆☆ |
| Miniero (2004),  Italy.^18^ | Multicenter retrospective study. | 68 infants: Kidney: 52 infants.  Liver: 7 infants.  Heart: 8 infants  (1 twin). | 16 women: CsA + AZA + steroids,  15 women: CsA,  14 women: CsA + steroids,  8 women: AZA + steroids,  2 women: TAC + steroids,  1 woman: AZA + TAC + steroids,  1 woman: CsA + AZA. | Mean gestational age: 36.1 (range: 22-40)  28 (41.8%) preterm | 2485 (range: 1150-4000).  6 infants: IUGR. | Follow up ranging from 2 months till 13 years.  Retrospective questionnaires, patient record data and interviews in person or by telephone. Interview questions regarding:   - growth, - vaccinations, - allergic reactions, - diseases, - laboratory tests, - last measured height and weight. | - None of the infants had side-effects of vaccinations. - Development uneventful, growth regular. - Conditions present at birth completely resolved. | Selection: ☆☆☆  Comparability: - Exposure: ☆ |
| Bar (2003),  Israel.^63^ | Single center retrospective cohort study.  Control group: women with underlying renal disease with no Tx nor immunosuppressive drug treatment. | Kidney:  48 infants from 38 women.  Control: 48 infants from 41 women. | 24 women: Prednisone + CsA + AZA,  6 women: prednisone + CsA,  3 women: prednisone + TAC,  4 women: prednisone + AZA,  1 woman: prednisone + AZA + TAC.  (AZA 2–3 mg/kg/day, prednisone 0.15 mg/kg/day, CsA 4 mg/kg/day) | 29 (60%) preterm  Control: 10 (21%) preterm  P=0.001 | 25 (50%) IUGR  Control: 8 (17%) IUGR  P = 0.001 | 2-7-years follow-up.  Retrospective analyses of medical records.   - Assessment of short term outcomes e.g. caesarean delivery, hospitalization, stillbirths.   Blinded periodical examination up to 7 years:   - maternal renal function, - infant status (presence of severe handicap). | - No significant differences in major malformations was found between the groups (4.2% (n=2) in both groups). This is similar to what is reported in the general population (2-3%). - No significant differences in the rate of mild errors of morphogenesis were found. (20.8% (n=10) in the Tx group and 16.6% (n=8) in the control group). - No significant difference in the rate of severe disability in the long term was found. 8% (n=3) in the Tx group (2 cases of cerebral palsy due to extreme prematurity and 1 was deaf, probably due to a CMV infection) and 2.4% (n=1) in the control group. | Selection: ☆☆☆☆ Comparability: ☆ Exposure: ☆ |
| Sgro (2002),  Canada.^31^ | Retrospective cohort study.  Control group matched to maternal age and smoking status. Control group exposed to non-teratogenic agents. | Kidney:  32 infants in 26 women (44 pregnancies).  Control: 88 infants. | 26 infants: CsA + AZA + prednisone.  13 infants: AZA + prednisone.  5 infants: CsA + prednisone. | Mean: 36.5 +/-2.7  Control: 40.2 +/- 1.6  p<0.0001 | Mean: 2540 +/- 670  Control: 3590 +/- 530  p<0.0001 | Follow up mean 3.1 year (range 3 months till 11 years).  Retrospective analyses of medical records.  Pediatric follow up with a clinic visit:   - physical examination including growth parameters, - neurodevelopmental assessment with the Denver Developmental Screening test.   If a clinic visit was not possible: telephone interview concentrating on growth, general health outcomes and developmental history using the Denver Developmental Screening test was performed (n=16). | - The parameters of the control group were all within the normal limits of the general population of Toronto.   In the Tx group:   - Normal growth and development. - On follow-up, the Tx group’s weight for age was significantly higher and the length for age was significantly lower compared to the control group (68.9 +/- 30^th^ centile vs 54 +/- 30^th^ centile, p= 0.026 and 48 +/- 34^th^ centile vs 69.8 +/- 28^th^ centile, p = 0.005 resp.). - 1 child with insulin dependent diabetes mellitus. 2 children with asthma and 1 child with recurrent otitis media. - Developmental follow-up revealed 3 cases of developmental delay not related to asphyxia: 1 child with moderate to severe sensorineural hearing loss requiring a hearing aid, 1 child with a learning disability and 1 child with pervasive developmental disorder. | Selection: ☆☆☆☆ Comparability: ☆ Exposure: ☆ |
| Giudice (2000),  France.^32^ | Single center prospective cohort study. | 14 infants: Kidney: 10 infants (1 twin).  Pancreas-kidney: 1 infant.  Heart:  2 infants.  Liver: 1 infant. | All women: CsA (average blood trough level 234 +/- 115 (range 121-511) ug/l). | Mean: 34 +/- 3 weeks | Mean: 2018 +/- 620 (range 1160-3280) | Renal function tests performed in 12 children at 2.6 +/- 1.8 years (range 1.0-6.9 years):   - blood pressure, - inulin clearance, - paraminohippuric acid clearance, - microalbuminuria, - electrolyte reabsorption rate, - renal ultrasound including renal size.   All 14 children:   - retrospective neonatal history, - complete physical examination at the time of the renal function study. | - 2 children excluded due to the diagnosis of hereditary nephritis (1 Alport syndrome, 1 familial dominant focal segmental glomerulosclerosis). - Body weight 12.5 +/-4.2 kg, height: 88.9 +/- 14.8 cm. - 9 of the 12 children had normal BMI, the remaining 3 children were in the low range. - All 12 children had normal psychomotor development. - Blood pressure: 94 +/-7 / 55 +/-5 mmHg, - inulin clearance: 117 +/- 28 ml/min/1.73 m2, - paraminohippuric acid clearance: 545 +/- 124 ml/min/1.73 m2, - filtration fraction 0.23 +/- 0.03, - microalbuminuria: 4.2 +/- 3.5 mg/mmol. - Electrolyte tubular reabsorption rates and urine concentrating capacity normal, - plasma concentration of sodium, potassium, chloride, calcium and phosphate normal. - No child had proteinuria. - Renal ultrasound normal. | Selection: ☆☆☆ Comparability: - Exposure: ☆☆ |
| Willis (2000),  United Kingdom.^33^ | Single center cross-sectional prevalence study. | Kidney:  48 infants (23 girls) from 34 women  (1 triplet). | 1 woman: prednisolone,  1 woman: prednisolone + AZA + CsA,  16 women: AZA + prednisolone,  16 women: CsA + prednisolone. | Mean not described.  27 (56%) preterm. | Mean not described.    21 (44%) LBW, 21 (44%) SGA. | Median follow up: 5.2 years (range 9 months - 18 years).  Information obtained via surveys, semi-structured interviews, medical records and by physical examination carried out by 1 of the 2 researchers.  Physical examination:   - standard mercury sphygmomanometry. Values were converted to age related centiles using values from the United States task force on children’s blood pressure. - Developmental assessment considering the developmental milestones, scholastic and educational achievements. - Routine analyses of a single urine sample and if abnormal microscopic and bacteriological examination. - Ultrasound examination of the urinary tract. | - Normal general health in 94% (n=45). 2 children had asthma and 1 had recurrent respiratory infections. - Physical assessment unremarkable in 95% (n=41). 1 child with cerebral palsy (1 of the triplet) and 1 with a claw deformity. - Median height on the 50^th^ percentile (mean 43^rd^ centile, range: 3-97), - median weight on the 50^th^ percentile (mean: 44^th^ centile, range 5-97). - 3 subjects had height and/or weight below the 10^th^ percentile, which is within the normal distribution of the general population. - 44% was born with a birth weight <10^th^ percentile but they showed impressive catch up growth. - Development considered normal in 98%. (n=47). 1 child had a developmental delay (the child with cerebral palsy). - Urine analysis was abnormal in 2 children. These 2 sisters both had dysplastic kidneys with known renal impairment. - Median blood pressure on the 50^th^ centile (mean 52, range 25-97). No significant associations between BP and BW, gestational age, IUGR or weight at follow up were found with linear regression. - 4 out of 40 (10%) had urinary tract abnormalities on ultrasound which is significantly more than the general population (2.9%) (p=0.036). 1 child had a pelviuretric junction obstruction, 1 child a unilateral scar and 2 children (siblings) unilateral renal dysplasia. | Sample frame: yes Participants samples: yes  Sample size: yes Subjects and setting: no Data analysis: yes Valid methods: yes Valid measurements: yes Statistical analysis: yes Response rate: yes |
| Stanley (1999), United States.^56^ | Retrospective cohort study of the NTPR database together with interviews. | Kidney: 175 infants (52% girls) from 133 women. | All (n=133) of the women used CsA. No further data mentioned. | Mean 36 +- 3 weeks | Mean 2499 +- 765 | Range of the child’s age at interview: 4 months-12 years, mean age: 4.4 years.  Children were divided into three groups by  age:   - Group 1: 1 year - Group 2: 1 to 5 years - Group 3: 5 to 12 years   Developmental status of groups 1 and 2 was assessed using the Child Development Review system (CDR). The CDR provides  information on the child’s status in five areas of development: self-help, social, gross motor, fine motor, and language. Delays were identified if the child’s behavior in any area was ≥30% below  expected behavior for age. Children in group 3 were noted to have prior developmental or present educational morbidity if their mother reported their participation in developmental therapy programs as preschoolers or any of the following: grade retention,  requirement for tutoring or individual educational programming in  school, or treatment with medication for attention deficit/hyperactivity  disorder (ADD/HD) | - In 84% of the children no   developmental delay was found.   - Twenty-nine children (16%) had delays or needed educational support. Of which 1 (0.5%) was in group 1, 11 (6.3%) in group 2 and 17 (9.7%) in group 3)   For these 29 children, mean gestational age at birth was 34 weeks, with 14 (48%) ≤33 weeks.   - 18 children had more than one area of delay. - 3 (1.7%) of the 175 children had major disabilities. - Specific delays were identified infrequently in group 1, with   group 2 exhibiting a higher incidence of delays in all areas  of development, particularly language.   - Of the children in group 3, 13 (18%) required early intervention as preschoolers, 10 (14%) required educational support, 8 (11%) treatment for ADD/HD and 2 (3%) had grade failure. | Selection: ☆☆☆ Comparability: - Exposure: ☆☆ |
| McGrory (1998),  United States.^19^ | Retrospective NTPR database summary. | Combined pancreas -kidney and 1 pancreas followed by kidney:  20 infants from 18 women (23 pregnancies)  1 pregnancy with IVF. | All women: CsA based.  In 15 pregnancies: CsA + prednisone + AZA.  5 pregnancies: CsA and prednisone.  In 3 pregnancies: CsA + prednisone + AZA. | 34.8 +/- 2.4 (range 29-37), 14 (70%) preterm. | 2041 +/- 550 (range: 964-2807), 14 (70%) LBW, 4 (20%) VLBW. | Follow-up ranging from 1 month to 8 years.  Data collected from a questionnaire, medical records and telephone interviews.   - No specific long term outcome measurements. | At follow-up, all children were healthy with normal development.   - 1 child had an atrial septal defect which resolved at 1 year. | Selection: ☆☆☆ Comparability: - Exposure: - |
| Wu (1998),  Germany.  ^34^ | Retrospective cohort study. | Liver :  23 infants (1 twin) (10 girls) in 16 women. | 5 pregnancies: CsA + AZA + prednisolone,  6 pregnancies: CsA,  2 pregnancies: TAC + prednisolone,  2 pregnancies: TAC,  7 pregnancies: CsA + Prednisolone.  1 woman switched from TAC to CsA during pregnancy. | Mean: 38.6 +/- 2.2 (range 33-41).  3 preterm. | Mean: 2876 +/- 589.3 (range: 1950-4460).  1 SGA, 1 LGA. | Follow up range 1-99 months. 5 children <1 year at last follow up.  Data obtained via medical records and questionnaires.  Children examined by a pediatrician: postpartum, between 3-10 days, between 4-6 weeks, between 6-7 months, between 10-12 months, between 21-24 months, between 43-48 months.   - Height and weight, - psychological development, - neurological development.   Postal questionnaire evaluated by the pediatrician:   - psychological and psychosocial development. | - No unusual infections. - Even though the birth weight was low, the subsequent height and weight development appeared within the normal range. - Adequate psychological development. - No signs of the maternal liver disease observed in any of the children. | Selection: ☆☆☆ Comparability: - Exposure: ☆☆ |
| Jain (1997),  United States.^35^ | Prospective cohort study. | Liver:  27 infants in 21 women (2 infants died shortly after birth, long term follow up n=25). | TAC in all women (TAC doses were modestly increased during 5 of the 27 pregnancies and decreased in 6).  In six women also prednisone (daily doses of 5 (n=3), 7.5 (n=2), or 10 mg/day (n=1)).  1 woman: also AZA (50 mg/day). | Mean 2638 +/- 781  52% (n=14): preterm | Mean 36.6 +/- 3.3 | Multiple, frequency and timing of the follow-up moments not specified. Median follow-up of 39 months (range 10-76 months).  Prospectively collected data by patients, obstetricians and the physicians:   - weight for age percentiles calculated from the National Center for Health Statistics percentiles. | - All 25 babies had satisfactory postnatal growth and development with a mean weight percentile of 62+/-37 (median 80). - Some of the scores reflect the degree of prematurity and low weight at birth. 5 babies below the 10^th^ percentile. - Both parents of the only infant with a weight <5 percentile are of short stature (mother=4'11", father=5'6"). - 10 babies have percentile growth >95. | Selection: ☆☆☆ Comparability: - Exposure: ☆ |
| Wong (1995),  New-Zealand.^55^ | Single center retrospective cohort study. | Kidney:  11 infants in 9 women. | 4 women: prednisone + AZA,  5 women prednisone + AZA + CsA. | 3 (29%) preterm | 7 (45%) IUGR | Follow-up ranging from 15 months to 18 years.  Retrospective information from medical records:   - assessment of the clinical and laboratory data of the child in the pediatrics department, - physical growth, physical examination, - School performance, work achievement, - social behavior, - developmental milestones tested with the Denver developmental screening test. | - 1 infant died of sudden infant death syndrome ate age 4 months. - The others (n=10) had normal physical growth, physical examination and developmental milestones. - School performance, work achievement and social behavior within the normal limits. - 1 child had a minor physical abnormality involving the abductor policies brevis tendon. | Selection: ☆☆☆ Comparability: - Exposure: ☆ |
| Pilarski (1994),  Canada.^45^ | Cross-sectional cohort study.  Control group: age-matched unexposed children. | Kidney: 11 infants from 9 women.  Liver:  1 infant. | 2 women: CsA + AZA + prednisone,  5 women: CsA + prednisone,  4 women: AZA + prednisone. | Term, 35, 36.5, term, term, term, 29.5, term, term, 30, 30.  Liver child: not described.  Control: not described. | 3700, 1750, 2760, 2840, 2760, 2400, 1290, 2860, 2730, 930, 930.  Liver child: not described.  Control: not described. | 1 follow up per infant. Follow up time ranging from 5 months till 9 years (1 child <1 year at follow up).   - Venous blood samples of 10 KTx children, - cord blood sample from1 KTx child, - blood from 4-6 months old baby’s in the control group, - peripheral venous blood from 1 LiTx child at age 8 years.   Immunological assessment of blood samples of 8 children:   - Quantity of immunoglobulins, complement and autoantibodies to various tissues and cellular components, - monoclonal antibody staining for: CD20, CD5, CD45 isoforms RA and R0, - antibody response to childhood vaccinations: polio, diphtheria, tetanus, measles, mumps and rubella. | - No clinical signs of immunodeficiency. No opportunistic infections, weight loss, growth retardation, chronic febrile illnesses, arthralgias or diarrhea were found. - Proportion CD20+ B cells in the CsA exposed children was within the range of the control group with the exception of one 5 months old baby. - The percentage of CD5+ B cells was in both the CsA and the control group 60-75%. - Normal proportions of CD3+, CD4+ and CD8+ T cells where found in the CsA group and the AZA group compared to the control. - CsA exposed children had significantly more CD45RA+R0- T cells compared to the control group, suggesting that with CsA T cell development appears to be delayed. - AZA exposed children had significantly more CD45RA-R0+ T cells compared to the control group, suggesting that with AZA T cell development appears to be accelerated. - In CsA exposed children of 1 year or older lower expression of CD29 on T cells compared to the control group. - Significantly higher expression of CD29 on T cells in the AZA group. - Quantitative immunoglobulins and complement levels within normal limits. - Most CsA children made protective antibodies against the vaccinations. - No autoantibodies were found except for 2 children who made antiRo/SSA antibodies although their mother was anti-Ro-negative. - All 10 children have developed normally except for 1 child who has cerebral palsy and is mildly mentally impaired. | Inclusion criteria: yes Study subjects: yes Exposure measured: yes Measurements objective: yes Confounding factors: no Strategies against confounders: no Outcome measurements: yes Statistical analysis: yes |
| Pahl (1993),  United States.^43^ | Retrospective cohort study. | Kidney:  26 infants (11 girls) in 21 women. | 1 woman: prednisone,  15 women: prednisone + AZA,  2 women: prednisone + AZA + CsA,  4 women: prednisone + CsA. | Mean: 36.25, median: 38 (range 32-41).  9 preterm | Mean: 2685 +/- 697.3 | Mean follow up: 5 years, range: 1 week - 18 years (5 children <1 year).  Analyses of medical records, interviews with the physician:   - medical records of the mothers, - medical records of the children from the pediatrics department if present.   Interviews of the mothers by telephone or email:   - outcome of pregnancy and the childhood development of their child(ren).   Developmental information on 10 children available at 6 months, 1 year and 2 years. | - All children reported as healthy by their parents and their pediatrician except for 1 child with a malignant hepatoblastoma with pulmonary metastases at age 18 months (treated and now well). - Children normal in all developmental milestones. - Body weight remained below the 25th percentile in 4 children (1 at 1 year, 3 at 2 years). - Body weight remained below the 50^th^ percentile in 4 children (3 at 6 months, 1 at 2 years). | Selection: ☆☆☆ Comparability: - Exposure: ☆ |
| Shaheen (1993), Saudi Arabia.^59^ | Cross-sectional cohort study | Kidney: 26 infants | All mothers CsA. | 64% preterm birth | Not described. | Mean follow-up 39 months (range 6-72 months)   - Basic tests of kidney function and integrity in 22 children - Serum cyclosporine was measured in whole blood using radioimmunoassays | - Mean weight 13.8 kg (range2.6-26.4), mean height 89.7 cm (range 21-119). - Renal function and integrity were found to be normal. - Mean urea: 4.9 mmol/L (range 3-7.8), - Mean creatinine: 35 umol/L (range: 22-48), - Mean chloride: 108 mmol/L (range: 104-113), - Mean phosphate: 1.65 mmol/L (range: 1.63-1.88), - Mean bicarbonate: 20.4 mmol/L (range: 18-23), - Mean potassium: 4.5 mmol/L (range: 3.8-5.4). - Urinary aminoacid chromatography was normal in all 8 children tested. - RBC morphology was normal in all 12 children tested. - Urinary dipstick was negative for blood and ranged between negative and trace for protein in the 13 children tested. - The mean cord blood CsA levels was 149.5 ng/L (range 98-263) tested in 5 children, whereas the CsA blood level in the last trimester in the maternal blood was 320 ng/L (range 109-511) . | Sample frame: yes Participants samples: no Sample size: yes Subjects and setting: no Data analysis: yes Valid methods: yes Valid measurements: yes Statistical analysis: no Response rate: yes |
| Wagoner (1993),  United States.^25^ | Retrospective survey study. | 31 infants (2 twins) in 26 women.  Heart: 28 infants.  Heart and lung:  3 infants. | 16 women: CsA + AZA + corticosteroids,  3 women: CsA + corticosteroids,  3 women: CsA + AZA,  1 woman: CsA,  3 women: unknown.  In 11 women the CsA dose was increased during pregnancy by 13%-227%.  In 6 women the CsA levels were lowered.  In 4 women increased corticosteroid doses were given because of rejection.  3 women stopped AZA (2 because of cholestatic jaundice, 1 because of severe anemia). | 41% (n=12) preterm | 17% (n=5) LBW | Mean follow up 3.4 years (range 3 months till 6.5 years).  Questionnaires study:   - no specific long term outcome measurements described. | - No adrenal insufficiency, neonatal infections or teratogenicity. - All children are reported in good health. | Selection: ☆☆ Comparability: - Exposure: ☆ |
| Rasmussen (1981),  Sweden.^47^ | Prospective cohort study. | Kidney:  5 infants (2 girls) in 4 women. | 4 women: AZA (100-150 mg/day) + prednisolone (4-6 mg/day),  1 woman: AZA (100-150 mg/day). | 38, 34, 37, 34, 38 | 2800, 2130, 2830, 2470, 2840 | Follow up ranging from 4.5-9 years. Follow up frequency between 2 and 4 times.  Multiple follow up moments at the local pediatric clinic:   - somatic and psychomotor evaluation at regular intervals.   Immunological follow up from peripheral blood at multiple time points:   - percentage rosette-forming PBM’s (T cells, normal range 50-70%), - proliferative responses of PBM to phythemagglutinin and pokeweed mitogen, - counting the PBMs with surface immunoglobulins using fluoresceinated anti-light chain antisera (B cells, normal range 10-20%, last follow up: 3-5%), - quantitative immunoglobulin levels for IgG, IgA and IgM, - serum testing for antibodies against hepatitis B, polio virus, Haemophilus influenza and Escherichia coli. - Serum aspartate transferase and alanine transferase in HBsAg-positive children. - Chromosomal analyses was performed in 4 children. | - 4 children have shown completely normal somatic and psychomotor development. - 1 child has a cardiac anomaly. Findings at clinical examination, phonocardiography, ECG and X-ray suggest a mild combined valvular aortic anomaly, possibly in combination with a ventricular septal defect. - 1 child has a slightly delayed and very uneven psychomotor development with marked clumsiness and articulatory dyspraxia but is not mentally retarded. - No chromosomal abnormalities were observed. - No significant infectious problems were observed. Common viral respiratory infections and uneventful courses and there was no unusual number of bacterial complications. - 1 child suffered from acute bacterial pyelonephritis twice. - IgG, IgA and IgM levels normal, - percentage T and B cells normal, - proliferative response to mitogens of the lymphocytes normal, - antibodies against E. coli and H. influenza present. - 1 child had granulocytopenia varying between 0.54 and 1.00x10^9^ neutrophil cells/L without infectious problems. His neutrophil count normalized after measles to 2x10^9^. - 3 children whose mothers were chronically HBsAg-positive became carriers and all had serum HBs and HBe antigens, and HBs antibodies. | Selection: ☆☆☆ Comparability: - Exposure: ☆☆ |
| Korsch (1980), United States.^11^ | Retrospective cohort study. | Fathers with a KT: 4 infants (0 girls) from 3 fathers  Mothers with a KT: 6 infants (2 girls) in 5 women | No data. | Father KT: 34-36 and n=3 were term  Mother KT: 34, 36 and n=4 were term | Father KT: 2041, 3345, 2693, 2932. No SGA.  Mother KT: 3052, 2010, 2410, 4130, 2810, 3232. No SGA. One was large for gestational age. | Follow-up: ranging from 4 months to 6 years and 8 months (father KT: 4 months, 10 months, 11 months, 2 years 7 months and mother KT: 7 months, 1 year, 1 year 10 months, 2 years 4 months, 3 years 2 months, 6 years 8 months).  Data from:   - Patient records - Physical examination by a pediatrician - Developmental evaluations on nine of the children were performed by a specialist in assessing child development. - The Stanford-Binet test was administered to the two oldest children. - The seven children under 30 months of age were evaluated with the Gesell Developmental Schedules and the Bayley Scales of Infant Development. - The remaining child was evaluated only with the Bayley Scales by a child psychologist. - A semistructured interview based on questionnaires developed for use in well-child clinics and in a child development project was carried out with the eight recipients. The interview was designed to elicit parents' attitudes about their child's development. - All parents were interviewed by a social work assistant trained in sociologic research methods. | - Health during the first year of life was excellent in all offspring. - No unusual intercurrent illnesses, metabolic disorders, or other physical problems were described. - There were no clinical indications of adrenal or immunologic dysfunction. - All ten offspring were physically in good health at the time of follow-up. - The presence of sickle cell trait in 1 infant was expected. - Physical growth had been normal in all the infants. The three children who were below the 25^th^ percentile for weight at the time of follow-up, weighed 2010, 2410, and 2693 gram at birth. - All children tested within the range of normal for the developmental assessment. - The interview with the families revealed that the majority of children are developing without perceived behavioral problems, and their upbringing is not being affected by the allograft recipient status of their parents. - Five of the parents did report a tendency toward seeking immediate medical attention for self-limiting illnesses in their children, which was based on a philosophy of taking no chances. | Selection: ☆☆☆ Comparability: - Exposure: ☆ |

Bias appraisal tool: prospective and retrospective cohort studies: Newcastle Ottawa Scale (NOS) for cohort studies, for cross-sectional prevalence studies the Joanna Briggs Institute (JBI) for prevalence data cross-sectional studies and for cross-sectional analytical studies the JBI for analytical cross-sectional studies. Abbreviations: AZA: azathioprine, BCG: Bacille Calmette Gueri, BMI: Body Mass Index, CBCL: Child Behavior Checklist, CES-D: Center for Epidemiologic studies Depression Scale, CI: confidence interval, CNI: calcineurin inhibitor, CsA: cyclosporine A, ELISA: Enzyme-Linked Immuno Sorbent Assay, GS: glucocorticosteroids, HTx: heart transplantation, LGA: large for gestational age, Ig: immunoglobulin, IPTR: international pancreas transplant registry, IUGR: intra uterine growth restriction, IVF: In vitro Fertilization, IQ: intelligence quotient, JBI: Joanna Briggs Institute, KTx: kidney transplantation, LBW: low birth weight, LiTx: liver transplantation, MMF: mycophenolate mofetil, NOS: Newcastle Ottawa Scale, NTPR : National Transplantation Pregnancy Registry, PBM: peripheral blood mononuclear cells, SES: socioeconomic status, SGA: small for gestational age, TAC: tacrolimus, Tx: transplantation, UK-WHO: United Kingdom World Health Organization, VLBW: very low birth weight, VMI-4: Beery Developmental Test of Visual Motor Integration, WAIS-III: Wechsler adult intelligence scale (WAIS-III), WASI: Wechsler Abbreviated Scale of Intelligence, WISC-IV: Wechsler Intelligence scale for children, WPPSI: Wechsler Preschool and Primary Scale of Intelligence, WPPSI-R: Wechsler Preschool and Primary Scale of Intelligence-Revised, WRAVMA: Wide Range Assessment of Visual Motor Abilities. Definitions: preterm: <37 weeks of gestation, extremely preterm: <32 weeks of gestation, LBW: <2500 gram, VLBW: <1500 gram, IUGR: weight < 10th percentile for the gestational age, LGA: weight > 90th percentile for the gestational age

**Table S2B. Summary of the included studies**, **case reports**

| **Author (Year),**  **country** | **Transplanted organ, number of children** | **Immunosuppressive regimen** | **Gestational age (weeks)** | **Birth weight (gram)** | **Outcome measures** | **Long-term outcome** | **Bias appraisal score** |
| --- | --- | --- | --- | --- | --- | --- | --- |
| Rao (2019), Australia.^41^ | Kidney: 1 infant. | TAC + prednisolone +AZA  TAC: 5 ng/mL | 34 | 2350 | The weight of the infant was followed up for 2 years. | - Weight of the infant remained normal. | Demographics: yes History: no Current condition: no Assessment methods: no Intervention/treatment: yes  Post-intervention condition: N.A. Adverse events: N.A. Takeaway lessons: no |
| Mahmoud (2017),  Kuwait.^15^ | Kidney:  4 infants (1 triplet) (4 males)  Clomiphene citrate used to induce ovulation in the triplet pregnancy. | AZA + prednisolone + TAC. | 32, 32 (triplets) | 1400, triplets: 1320, 1380, 1275 | No specific outcome measurements described.  Follow up at birth, discharge, 12 months and 24 months. | - Triplets: normal mental and physical growth at follow up. - Weight at 24 months: 9, 16, 11 kg. - One child developed asthma. | Demographics: yes History: no Current condition: no Assessment methods: no Intervention/treatment: yes Post-intervention condition: N.A. Adverse events: N.A. Takeaway lessons: no |
| Kociszewska-Najman (2012),  Poland.^29^ | Liver:  2 infants in 2 women. | 1 woman: CsA.  1 woman: CsA + prednisone. | 38, 39 | 3100, 2960 | Case report 1.  During a follow up visit at 7 months:   - length, weight, head circumference, - neurodevelopmental and socio-emotional assessment, - mental ability tested with the Cattell Infant Intelligence scale.   Case report 2  Follow up visit at 21 months:   - length, weight and head circumference, - blood pressure, - laboratory tests: full blood count, electrolytes, glucose, cholesterol, triglycerides, aminotransferases, urea, creatinine and urine test, - IgA, IgM and IgG serum concentration, - abdominal ultrasound and echocardiogram, - psychomotor development assessment, - mental ability tested with the Cattell Infant Intelligence scale. | Case report 1, follow up at 7 months:   - Normal physical development: length 90 percentile, weight 97 percentile and head circumference 75 percentile, - neurodevelopment and socio-emotional development age-adequate, - IQ 130.   Case report 2, follow up at 21 months:   - Normal physical development: height 50 percentile, weight 10^th^ -25^th^ percentile, head circumference 25^th^-50^th^ percentile, - blood pressure 105/55 mmHg, - laboratory tests normal, - normal IgA and IgM concentration. IgG concentration was below the normal range. - Normal abdominal ultrasound and echocardiogram, - mental ability was assessed at an IQ of 108 but the child was unwilling to cooperate. | Demographics: yes History: no Current condition: yes Assessment methods: yes Intervention/treatment: yes Post-intervention condition: N.A Adverse events: N.A. Takeaway lessons: no |
| Nicovani (2009),  Chile.^27^ | Kidney:  3 infants (triplet).  Gynecologic treatment used to induce ovulation. | CsA + AZA + prednisolone | 28 | 840, 860 and 1020 | No specific long term outcome measures described.   - 4 year follow up. | - Normal psychomotor and physical development. | Demographics: yes History: no Current condition: no Assessment methods: no Intervention/treatment: yes Post-intervention condition: N.A Adverse events: N.A. Takeaway lessons: yes |
| Xia (2008),  China.^17^ | Liver: 1 infant (boy). | CsA.  Patient discontinued CsA in the 1st trimester afterwards hospitalized because of suspected rejection. Treated with MMF 1 g/day+ CsA 450 mg/day for 2 months after that again CsA alone. | 37 | 2000 | 4 year follow up. Follow up every 3-6 months.   - Routine follow up visits, - patient self-examination of the baby’s growth and development. | - Normal growth and development in the 4 year follow up. | Demographics: yes History: no Current condition: no Assessment methods: yes Intervention/treatment: yes Post-intervention condition: N.A. Adverse events: N.A. Takeaway lessons: no |
| Scott (2002), United States.^28^ | Kidney: 5 infants (3 girls) (1 mother) | Azathioprine 75 mg/day, prednisone 5 mg/day. | Not described | 3300, 3420, 2600, 3050, 2775 | Follow-up at one time-point, age of the offspring: 23, 21, 18, 17, 15 years.   - No outcome measurements described. | - No unusual health problems in 4 children - 1 child experienced transient joint stiffness at 8 years and ulcerative colitis was diagnosed at age 16 year. She had multiple auto-antibodies and SLE during pregnancy, pregnancies were characterized by the mid- trimester fetal death and early- onset preeclampsia. | Demographics: no History: no Current condition: no Assessment methods: no Intervention/treatment: yes Post-intervention condition: N.A. Adverse events: N.A. Takeaway lessons: yes |
| Morini (1998), Italy.^20^ | Heart: 1 infant (girl). | 330 mg/day CsA + 50 mg/day AZA. The dosage of cyclosporine was progressively increased until it was 405 mg/day shortly before delivery. | 37 | 2330 | Case report with a 14 month follow up.   - No specific long term outcome measurements. | - At 14 months the baby is doing well. | Demographics: yes History: no Current condition: no Assessment methods: no Intervention/treatment: yes Post-intervention condition: N.A. Adverse events: N.A. Takeaway lessons: yes |
| Roll (1997),  Germany.  ^36^ | Liver: 1 infant. | CsA (320 mg/day) (trough concentrations 150–220 mg/mL)  + prednisolone (5 mg/day). | 29 | 590 | Case report with a follow up of 2 years and 6 months.   - No specific long term outcome measurements. | - Despite a complicated neonatal course (cholestasis, hypoglycemia, and mild bronchopulmonary dysplasia), normal neurodevelopmental status and good catch-up growth up at 2 years and 6 months follow up. - Large hepatoblastoma at 2 years and 6 months. | Demographics: yes History: no Current condition: no Assessment methods: no Intervention/treatment: yes Post-intervention condition: N.A. Adverse events: yes Takeaway lessons: yes |
| Eskandar (1996),  Canada.^21^ | Heart:  2 infants (2 girls) in 1 woman. | CsA + prednisone. | 41, 38 | 3250, 2880 | Case report with a follow-up of >2 years in both children.   - No specific method of assessment of long term outcomes mentioned. | - The children are doing well. | Demographics: yes History: no Current condition: no Assessment methods: no Intervention/treatment: yes Post-intervention condition: N.A. Adverse events: N.A. Takeaway lessons: no |
| Morita (1996),  Japan.^37^ | Kidney:  8 infants (4 girls) in 6 women. | 3 women (5 infants): AZA + prednisolone,  3 women (3 infants): CsA + AZA + prednisolone. | Mean: 36 (range: 34-39).  4 infants preterm | Mean: 2486 (range 2050-2950).  2 infants: IUGR | Mean follow up: 4.1 years (range: 1 year till 11 years).  1-time point of evaluation.   - No specific method of assessment mentioned. | At last follow up:   - 1 of the infants with IUGR has not grown well and is mentally retarded and suffers from muscle weakness. - 7 children are healthy and within +/- 1.5 SD of the mean of the 1990 Japanese standard growth curve. | Demographics: yes History: no Current condition: yes Assessment methods: no Intervention/treatment: yes Post-intervention condition: N.A. Adverse events: N.A. Takeaway lessons: no |
| Liljestrand (1993),  Sweden.^64^ | Heart: 1 infant (girl) | Prednisolone (5 mg/day) + CsA (380 mg/day)  (CsA dose adjusted upwards to a maximum of 440 mg/day). | 40 | 2680 | Case report with 18 months follow-up.   - Specific long-term outcome measurements not described. - At 12 months: detailed evaluation at a regional specialized center in pediatric cardiology. | - Normal development up to 12 months. - At 12 months the child acquired serious cardiac insufficiency. Dilated cardiomyopathy with an enlarged ventricle was diagnosed. - Cardiac catheterization did not indicate a congenital malformation. - At 18 months the child was doing relatively well and on long-term medication (digitalis and diuretics). | Demographics: yes History: no Current condition: no Assessment methods: no Intervention/treatment: yes Post-intervention condition: N.A. Adverse events: yes Takeaway lessons: yes |
| Baarsma (1992),  Netherlands.^46^ | Liver: 1 (girl). | Prednisolone (10 mg/day) + AZA (125 mg/day) + CsA (320 mg/day 1st trimester, then tapered to 200 mg/day). | 38 | 2260 | Case report with a 2-year follow-up.   - 2 year follow-up, not clear how many follow-up moments. - Immunoglobulin assessment at birth, 4 and 24 months, - lymphocyte counts (CD3+, CD4+, CD8+ and CD20+) at 1, 10 and 24 months, - functional assessment of the immune system at 1, 10 and 24 months.   Comparison of lab values with:   - The normal range of Ig values in the hospital. - The normal levels of lymphocyte subpopulations according to a reference. | - Uneventful neonatal course, - normal renal function, normal blood counts and normal immunoglobulin levels at birth, - normal neurobehavioral development, - normal growth, - no functional defects of the immune system, no unusual infections. - Routine immunization with diphtheria, pertussis, tetanus, poliomyelitis, measles, rubella and mumps vaccinations were uneventful. - Immunoglobulin levels and IgG subclasses at birth, 4 and 24 months were normal for age. - Total T (CD3+), CD4+ and CD8+ and especially the total B (CD20+) cells were low at 1 and 10 months. - At 24 months the CD3+, CD4+ and CD20+ cells returned to the low normal range. CD8+ levels remained low. - Functional assessment of the immune system showed normal in vitro lymphocyte transformation after exposure to lectin, recall antigens and allogeneic cells. | Demographics: yes History: yes Current condition: yes Assessment methods: yes Intervention/treatment: yes Post-intervention condition: N.A. Adverse events: N.A. Takeaway lessons: yes |
| Grow (1991),  United States.^54^ | Liver:  2 infants (twins). | CsA (300 mg/day) + AZA (50 mg/day) + prednisone (15 mg/day).  After 10 weeks rejection: 2 times 1 gram injections of iv methylprednisone and increasing CsA to 400 mg/day, at 28 weeks another 1 gm methylprednisone injection. | 33 | 1003 and 1340 (both birth weights <2.5 percentile for their birth weight curve for singletons). | Case report with a neurodevelopmental follow up for 25 months.   - Unspecified neurodevelopmental follow up of 25 months. | - At birth: both children had asymmetrical growth retardation. - Language, cognition and social skills normal in both children. - Neurodevelopment normal in 1 child. - 1 child had some abnormalities of neuromuscular tone and fine motor skill. | Demographics: yes History: no Current condition: yes Assessment methods: no Intervention/treatment: yes Post-intervention condition: N.A. Adverse events: N.A. Takeaway lessons: no |
| Scantlebury (1990),  United States.^53^ | Liver:  20 infants (1 twin) (8 girls) in 17 women. | 2 women: AZA + prednisone,  13 women: CsA + prednisone,  1 woman: prednisone + CsA + AZA.  1 woman only taking AZA sporadically discontinued immunosuppression after knowledge of the pregnancy. | 39, 40, 41, 40, 28, 35, 38, 34, 39, 38, 38, 32, 38, 36, 35, 34, 34, 28, 35, 35 | 3660, 2400, 2550, 3150, 1220, 2400, 3090, 1590, 2020, 3050, 2550, 2100, 3210, 1690, 2870, 1020, 1330, 1450, 2500, 2330.  4 children: IUGR. | Case series.  Follow up ranging from 9 months till 12 years (n=16 >1 year).   - No specific long term outcome measurements described. | - All children have normal growth. - 15 of the 16 children > 1 year have normal physical and mental development. - 1 child has immature speech development at age 2.5 years. - 4 children below 1 year all have normal milestones so far. - No adrenocortical insufficiency or lymphopenia reported. | Demographics: yes History: no Current condition: yes Assessment methods: no Intervention/treatment: yes Post-intervention condition: N.A. Adverse events: N.A. Takeaway lessons: yes |
| Key (1989),  United States.^22^ | Heart: 1 infant (girl). | Prednisone (11mg/day) + AZA (150 mg/day). | 39 | 3278 | Case report with a follow up of 3 years.   - No specific long term outcome measurements described. | - Normal growth and development at 3 years. | Demographics: yes History: no Current condition: no Assessment methods: no Intervention/treatment: yes Post-intervention condition: N.A. Adverse events: N.A. Takeaway lessons: no |
| Preieto (1989),  Spain.^23^ | Kidney:  4 infants (2 sets of twins). | 1 woman: CsA,  1 woman: CsA + prednisone + AZA. | 35, 35 | 2380, 2000, 1850, 2060 | 1 case report follow up at 22 months and 1 at 8 months.   - No specific long term outcome measurements described. | - Normal stature-weight development at follow up in all infants. | Demographics: yes History: no Current condition: no Assessment methods: no Intervention/treatment: yes Post-intervention condition: N.A. Adverse events: N.A. Takeaway lessons: yes |
| Boner (1981),  Israel.^24^ | Kidney:  2 infants (twins). | Prednisone (10 mg/day) + AZA (AZA dose was reduced from 100 mg/day to 50 mg/day during pregnancy). | 38 | 2500, 2600 | Case report with a follow-up of 6 years.   - No specific long-term outcome measurements for the physical and psychological assessment described.   Immunological response examination at 8-10 months.  Cell mediated immunity examination by:   - lymphocytic transformation measurement with phytohemaglutinin, - estimation of the secretion of macrophage migration inhibition factor, - by the PPD skin test, - by delayed hypersensitivity skin tests. | - Clinical follow-up of the infants till the age of 6 years revealed normal physical and psychologic development. - Cell mediated immunity within normal limits in both siblings. | Demographics: yes History: no Current condition: no Assessment methods: yes Intervention/treatment: yes Post-intervention condition: N.A. Adverse events: N.A. Takeaway lessons: yes |
| Berant (1976),  Israel.^38^ | Kidney:  1 infant (girl). | AZA (150 mg/day) + prednisone (15 mg/day). | 36 (preterm) | 1950 (SGA) | Case report.  Multiple follow up visits: up at birth, 3 months, 5 months and 2 years.   - Lymphocyte counts: T cells, B cells, - antibody titers, - serum IgG, IgA and IgM levels. - At birth: chest x-ray for the thymic shadow. - Lymphocytic transformation by phytohemagglutinin at birth and 2 years. | - Height conform the 25th percentile. - Weight advanced from below the 3rd percentile at birth to above the 30th percentile at 2 years of age. - Physical and psychomotor development normal. - No adverse clinical responses to routine vaccination were observed. - Thymus dependent and thymus independent immune systems both normal in structure and function. - Lymphocyte counts and serum IgG, IgA and IgM levels within normal limits at all follow-up moments. IgG levels were at low normal range. | Demographics: yes History: yes Current condition: yes Assessment methods: yes Intervention/treatment: yes Post-intervention condition: N.A. Adverse events: N.A. Takeaway lessons: yes |
| Price (1976),  United Kingdom.^39^ | Kidney:  2 infants (1 girl).  Control: 54 infants. | 1 woman: AZA (150 mg/day) + prednisone (gradually reduced from 50 mg to 10 mg/day).  1 woman: AZA (gradually reduced from 150 to 50 mg/day) + prednisone (gradually reduced from 30 to 10 mg/day). | 36, 31  Control: not specified. | 1870, 1280  Control: not specified. | Case report 1: follow up of 32 months. Case report 2: follow up of 24 months.   - Not specified how many follow up moments. - No specific long term outcome measurements described. - Developmental tests not specified.   In both children: blood lymphocyte and cortisol levels were measured at multiple moments for 25 days.  In both children: chromosome analyses at birth, 6 months, 14 months, 20 months and 24 or 32 months. | 1 infant:   - In the neonatal period: unusual small thymic shadow on x-ray, - good catch up growth; height, weight and head circumference at the 45th percentile, - normal developmental tests.   1 infant:   - neonatal period: normal thymic shadow on x-ray, - at 24 months: slight bilateral indentation of the costal margins, - height, weight and head circumference at the 45th percentile. - Initially both infants had lower than normal lymphocyte counts and cortisol levels but levels quickly recovered. - Peripheral blood: chromosomal aberrations similar to aberrations present in the mother were found soon after birth and diminished over time. At last follow up the aberrations could no longer be identified. - Bone marrow: no chromosomal abnormalities were found in both infants. | Demographics: yes History: yes Current condition: yes Assessment methods: no Intervention/treatment: yes Post-intervention condition: N.A. Adverse events: N.A. Takeaway lessons: yes |

Bias appraisal tool: Joanna Briggs Institute (JBI) for case reports. Abbreviations: AZA: azathioprine, CNI: calcineurin inhibitor, CsA: cyclosporine A, GS: glucocorticosteroids, HTx: heart transplantation, Ig: immunoglobulin, IUGR: intra uterine growth restriction, IQ: intelligence quotient, KTx: kidney transplantation, LiTx: liver transplantation, MMF: mycophenolate mofetil, SGA: small for gestational age, TAC: tacrolimus, Tx: transplantation. Definitions: preterm: <37 weeks of gestation, extremely preterm: <32 weeks of gestation, IUGR: weight < 10th percentile for the gestational age.
